# CRISPR–Cas12a-mediated DNA clamping triggers target-strand cleavage

**DOI:** 10.1038/s41589-022-01082-8

**Published:** 2022-07-14

**Authors:** Mohsin M. Naqvi, Laura Lee, Oscar E. Torres Montaguth, Fiona M. Diffin, Mark D. Szczelkun

**Affiliations:** DNA–Protein Interactions Unit, School of Biochemistry, Faculty of Life Sciences, University of Bristol, Bristol, UK

**Keywords:** Single-molecule biophysics, Enzyme mechanisms, DNA metabolism, Single-molecule biophysics

## Abstract

Clustered regularly interspaced short palindromic repeats (CRISPR)–Cas12a is widely used for genome editing and diagnostics, so it is important to understand how RNA-guided DNA recognition activates the cleavage of the target strand (TS) following non-target-strand (NTS) cleavage. Here we used single-molecule magnetic tweezers, gel-based assays and nanopore sequencing to explore DNA unwinding and cleavage. In addition to dynamic and heterogenous R-loop formation, we also directly observed transient double-stranded DNA unwinding downstream of the 20-bp heteroduplex and, following NTS cleavage, formation of a hyperstable ‘clamped’ Cas12a–DNA intermediate necessary for TS cleavage. Annealing of a 4-nucleotide 3′ CRISPR RNA overhang to the unwound TS downstream of the heteroduplex inhibited clamping and slowed TS cleavage by ~16-fold. Alanine substitution of a conserved aromatic amino acid in the REC2 subdomain that normally caps the R-loop relieved this inhibition but favoured stabilisation of unwound states, suggesting that the REC2 subdomain regulates access of the 3′ CRISPR RNA to downstream DNA.

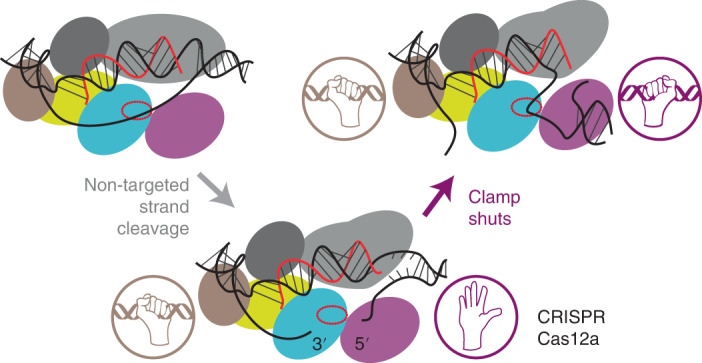

## Abstract (web summary)

Using single-molecule magnetic tweezers and biochemical methods, Naqvi et al. revealed how CRISPR–Cas12a regulates the DNA cleavage rate through a conserved stacking interaction between the R-loop and the W355 residue and the sequence of the CRISPR RNA 3′ end.

## Main

The clustered regularly interspaced short palindromic repeats (CRISPR)/CRISPR-associated (Cas) nuclease effectors (for example, type II-A Cas9 and type V-A Cas12a) evolved as prokaryotic defence systems against bacteriophages and are widely used in gene editing^1–3^. There is evidence that Cas12a (formerly known as Cpf1) has superior properties with fewer off-target events^4–6^, although this is contested^7^. Additionally, Cas12a has been exploited for nucleic-acid recognition diagnostics, including SARS-CoV-2 genomic RNA detection^8^. RNA-guided DNA recognition occurs by strand separation of a protospacer target to allow Watson–Crick base pairing between the DNA targeted strand (TS) and the spacer sequence of a CRISPR RNA (crRNA), and the unwinding of a non-targeted strand (NTS)^9^. Following this R-loop formation, type II Cas9 employs two nuclease domains, RuvC and HNH, to cut the NTS and TS, respectively^10,11^. By contrast, type V Cas12a has a single RuvC domain which cuts DNA in an obligatory sequential mechanism; NTS cleavage followed by TS cleavage^12–17^. However, it is unclear how RuvC transitions between cleaving the two strands^9^. Better understanding of this mechanism will aid design of Cas12a enzymes with improved properties.

**Fig. 1:**
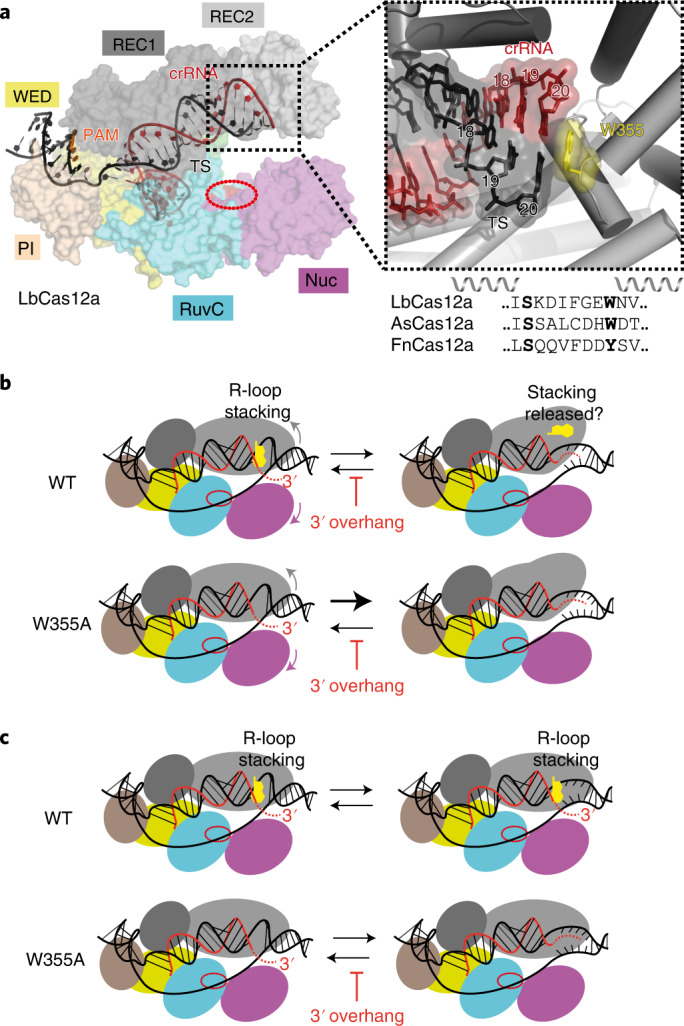
R-loop stacking by Cas12a and downstream DNA breathing during R-loop formation. **a**, Structure of LbCas12a (PDB: 5XUS^21^) with REC1 (dark grey), REC2 (light grey), Wedge (WED, yellow), PI domain (wheat), RuvC nuclease (cyan) with catalytic site (red oval) and Nuc (magenta) shown as protein surface, and DNA (black) and crRNA (red) as cartoons. The path of the NTS is not resolved in this structure. The inset shows the structure around the aromatic residue (W355 in LbCas12a) that stacks against the 20th base pair of the R-loop between the crRNA and TS. The amino acid sequence alignment of the aromatic residue loop is shown for LbCas12a, *Acidaminococcus sp*. BV3L6 Cas12a (AsCas12a) and *Francisella novicida* Cas12a (FnCas12a). **b**, Cartoon model of the REC2/Nuc domain motions^24^, which could move the aromatic residue from its R-loop stacking position and support PAM-distal DNA breathing. A W355A mutant removes the stacking interaction and would thus favour DNA breathing. A 3′ crRNA overhang complementary to the downstream TS (red dotted lines) could anneal and extend the R-loop during breathing, inhibiting R-loop dissociation. **c**, Alternative model where the REC2 subdomain/W355 do not move and the stacking interaction remains in place during breathing, preventing the 3′ crRNA overhang from annealing. A W355A mutant would allow annealing, favouring DNA breathing states and inhibiting R-loop dissociation.

**Extended Data Fig. 1:**
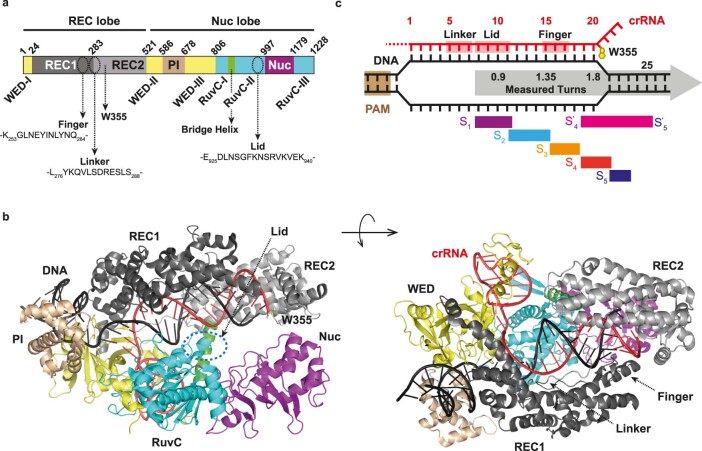
Domains, ternary structure, and key mechanistic elements of *Lachnospiraceae bacterium* ND2006 Cas12a. (**a**) Domain structure of *Lachnospiraceae bacterium* ND2006 (Lb) Cas12a, indicating REC1 and REC2 subdomains, the PAM-interacting domain (PI, wheat), the Wedge domain (WED, yellow), the RuvC nuclease (RuvC, cyan), the Nuc domain (purple) and the Bridge helix (green). The positions and amino acid identities of the ‘finger’, ‘linker’ and ‘lid’ from Stella et al.^14^ and the R-loop stacking residue (W355), are indicated. (**b**) Structure of LbCas12a (PDB: 5xus, TTTA PAM)^20^, indicating the locations of the elements from panel a; note that the ‘lid’ is not resolved in PDB: 5xus. During R-loop formation, a number of conformational checkpoint need to be passed to activate the RuvC nuclease^14^: the ‘linker’, a loop connecting REC1 and REC2 lobes, firstly interacts with the 5th to 7th nucleotides of the crRNA; the ‘lid’ loop then interacts with the 8th to 11th crRNA nucleotides; finally, the ‘finger’ (a REC1 helix) interacts with the 15th to 17th crRNA nucleotides; additionally, the bridge helix plays a role in stringency and coordination of conformation changes. (**c**) Summary of some of the R-loop states classified here alongside the crRNA contact positions of the linker, lid and finger from Stella et al.^14^.

Cas12a structures are bi-lobed monomers, with recognition and nuclease lobes^13,14,18–23^ (Fig. 1a and Extended Data Fig. 1). A Cas12a–crRNA complex scans a DNA target until the flexible pocket formed by the wedge (WED), REC1 subdomain and PAM-interacting domain (PI domain) interacts with a specific protospacer adjacent motif (PAM; 5′-TTTV-3′, where V = A/C/G). Upon DNA binding, the PI domain reduces its motion, while REC2 and Nuc motions are increased so that they move outward to help accommodate the TS in the RuvC active site^14,22–24^. Distortion of the PAM double-stranded DNA (dsDNA) leads to strand separation and R-loop formation within the recognition lobe, starting with the pre-structured 5′ end of the crRNA spacer (the ‘seed’)^16,20,22,25^. As R-loop formation progresses, conformational checkpoints need to be passed so the catalytic pocket is made available to bind any ssDNA^14,20,26,27^ (Extended Data Fig. 1). At least 17 bp of hybrid is required to satisfy the checkpoints with mismatches tolerated in the final 3 bp of the R-loop^6,13,17,25,28,29^.

Following RuvC activation, the liberated NTS is ideally placed to dock first into the RuvC active site (Supplementary Fig. 1). In some instances, the cleaved DNA ends can re-enter the active site, leading to end trimming^30^. TS cleavage is geometrically limited as the target site is downstream of the R-loop, ~25 Å from the active site and in an incorrect orientation for nucleophilic attack without rotation to match the NTS polarity^9,12,31^. A suggested model is that downstream dsDNA unwinds, and the released single-stranded TS is bent towards RuvC^14^ (Supplementary Fig. 1). Structures consistent with these molecular gymnastics have been observed for the related Cas12b and Cas12f complexes^32,33^. Cryo-electron microscopy structures, single-molecule fluorescence resonance energy transfer measurements and molecular dynamics of Cas12a additionally support an inward closing motion of the REC2 and Nuc domains following NTS nicking^14,24^.

More recently, Cofsky et al.^30^ demonstrated that downstream DNA including the TS cleavage site is subject to DNA breathing. Here we corroborated these findings in the absence of DNA cleavage by directly observing the dynamics of transient and reversible downstream unwinding events by *Lachnospiraceae bacterium ND2006* (Lb) Cas12a using a magnetic tweezers assay^16,34^. We also observed that the LbCas12a R-loop was highly dynamic and heterogeneous, with occasional reversible R-loop dissociation, as observed by others^14,15,17^.

It has been suggested that REC2 dynamics control conformational changes in Nuc, regulating TS loading into RuvC^13,32^. The R-loop is capped by a stacking interaction with a conserved aromatic amino acid in REC2 (for example, W355 in LbCas12a)^21^ (Fig. 1a). We speculated that this residue and the REC2 subdomain may have two potential roles: Downstream DNA breathing may be regulated by REC2 subdomain positioning (Fig. 1b). Releasing the stacking interaction of the aromatic residue by movement of the REC2 subdomain would favour DNA breathing; alternatively, the REC2 subdomain and stacking interaction remain in place during breathing (Fig. 1c). Mutation of W355 to alanine resulted in more frequent and extended downstream DNA breathing but only when the crRNA included a 3′ overhang complementary to the TS. This effect of the 3′ RNA was not observed with wild-type (WT) Cas12a, suggesting that the stacked aromatic amino acid stacking interaction remains in place, preventing annealing (Fig. 1c), as noted previously^20^. From ensemble endonuclease assays, crRNA with a 3′ overhang inhibited the WT TS cleavage rate by ~16-fold and this effect was relieved by the W355A mutation, or by removing the overhang or making it non-complementary to the TS. By following nuclease activity in the single-molecule tweezers assay, we also observed a torque-resistant clamping of the downstream DNA after NTS cleavage, which we suggest corresponds to Nuc interactions that guide the TS to the RuvC active site. This clamped state was inhibited by the crRNA 3′ overhang, resulting in slower TS cleavage but was again relieved by the W355A mutation or changing the 3′ end. We propose that REC2 and the aromatic residue control positioning of the 3′ end of the crRNA and following NTS cleavage, move position to release the stacking and allow downstream DNA unwinding and clamping necessary for TS cleavage.

## Results

### Observation of dynamic R-loops and downstream DNA breathing

**Fig. 2:**
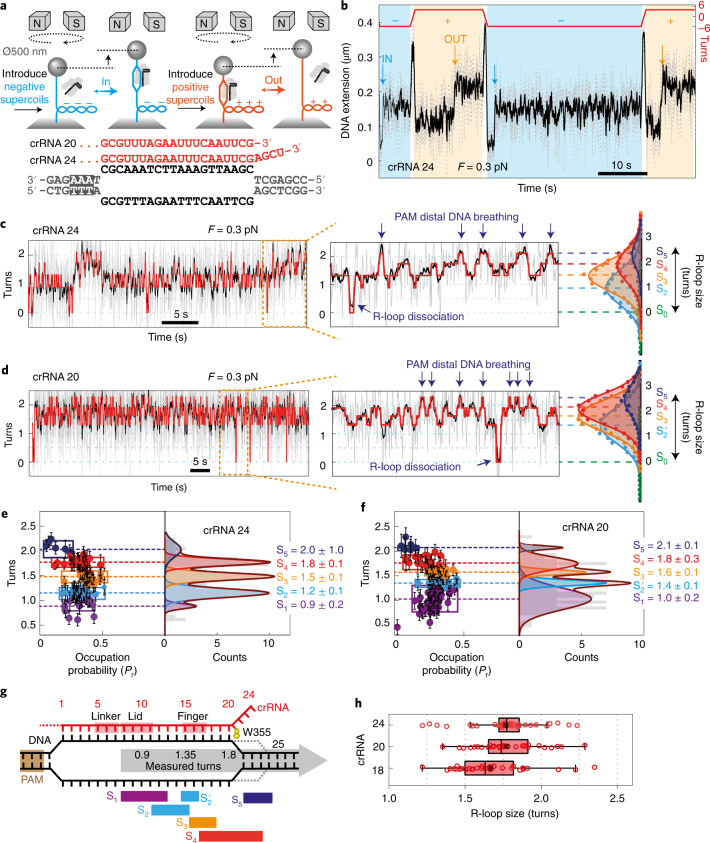
Wild-type Cas12a produces dynamic R-loop states in the absence of DNA cleavage. **a**, Representation of the magnetic tweezers experiments and DNA protospacer and crRNA spacer sequences. **b**, An example extension time trace in EDTA (to prevent DNA cleavage) for crRNA 24 (for all data: grey, 60 Hz raw data; and black, 10 Hz filtered) at 0.3 pN showing R-loop formation (IN, blue; at −7 pN nm) and dissociation (OUT, orange; at +7 pN nm) events. **c**, Example R-loop formation trace using crRNA 24 showing hopping between five states identified by HMM fitting (red); R-loop states S_2_, S_3_ and S_4_, and reversible transitions to an R-loop dissociated state (S_0_; Extended Data Fig. 3) and an extended state (S_5_; blue arrows). **d**, Example R-loop formation trace using crRNA 20 showing hopping between five states identified by HMM fitting (red). **e**,**f**, Plots for crRNA 24 (**e**) and crRNA 20 (**f**) showing the R-loop states identified by HMM analysis and their probability of occupation (*P*_r_) from multiple R-loop formation traces (*N* = 34 for crRNA 24, *N* = 30 for crRNA 20), with state positions confirmed from Gaussian fitting. Box width corresponds to the full width at half maximum of each peak. Filled circles (left) are mean values and error bars are standard deviation in turn positions measured from HMM fitting of individual traces (Methods). Errors in turn values (right) are standard deviation from the Gaussian peak fitting. **g**, Summary of the R-loop states classified from crRNA 24 and crRNA 20 alongside the crRNA contact positions of the linker, lid and finger^14^ (Extended Data Fig. 1). Dotted lines represent transient downstream breathing events that produce the extended state (S_5_). **h**, Box plots comparing main R-loop size measured from HMM analysis for crRNA 24 (*N* = 34), crRNA 20 (*N* = 30) and crRNA 18 (*N* = 33). Whiskers indicate 90% and 10% extreme values, the length of the box indicates interquartile range, the inner line is the median and the black small vertical bar is the mean of the population.

To observe real-time DNA unwinding, we used a single-molecule magnetic tweezers assay^16,34^ (Fig. 2a). A 2-kb linear DNA was tethered between a glass coverslip and a magnetic bead (500 nm diameter). DNA length was monitored by video microscopy of the bead^35^. A pair of permanent magnets above the flow cell stretched the DNA with fixed force and could be rotated to introduce positive or negative supercoiling. We initially used an ethylenediaminetetraacetic acid (EDTA) buffer to prevent DNA cleavage^16^. The crRNAs that are processed from Cas12a CRISPR arrays include a 4-nucleotide 3′ overhang that is not necessary for R-loop formation or gene editing^31^. We first compared WT Cas12a with crRNAs with or without this overhang (crRNA 24 versus crRNA 20) (Fig. 2a).

At 0.3 pN stretching force, introducing negative turns formed negative supercoils that shortened the apparent DNA length (Fig. 2b). Negative torque supports R-loop formation^16^, which resulted in a reduction in DNA supercoiling to balance the altered DNA linking difference, observed as an increase in DNA extension (IN events in Fig. 2a,b). To force the R-loop out, positive turns were introduced to generate positive supercoiling. Positive torque supports R-loop dissociation (OUT events in Fig. 2a,b). The process was repeated by cycling between negative and positive turns. Hereafter we convert DNA extension to DNA turns (Supplementary Fig. 2) to compare between different DNA molecules.

**Extended Data Fig. 2:**
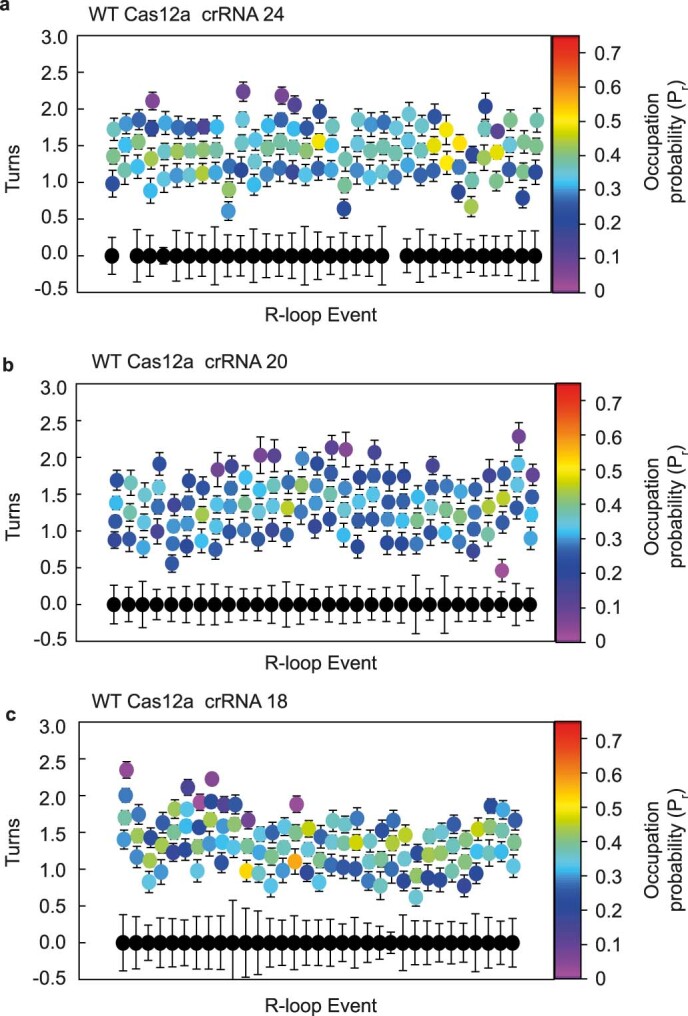
Size and dynamics of states for individual R-loop events at -7 pN nm using WT Cas12a. For each R-loop formation event using 1 nM WT Cas12a and either crRNA 24 (**a**, *N* = 34, 2 DNA molecules), crRNA 20 (**b**, *N* = 30, 2 DNA molecules) or crRNA 18 (**c**, *N* = 33, 1 DNA molecule), individual turn states were identified by HMM (filled circles, Materials and Methods). The Occupation Probability (P_r_) of the fitted states was calculated and is shown for each state by the heat map. Filled circles represent the mean values and error bars are s.d. of the measured turn size from HMM fitting of individual traces. In a few events in panel a, the R-loop dissociated state (black) could not be identified as the R-loop formed immediately upon turning to negative torque.

**Extended Data Fig. 3:**
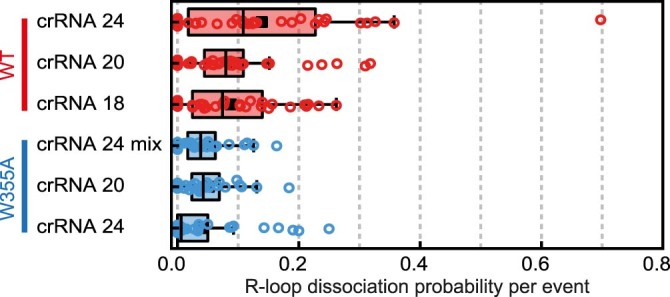
Probability of R-loop dissociation by wild type and W355A Cas12a. Box plots comparing R-loop dissociation event probability during R-loop formation at -7 pN nm measured from HMM analysis for WT Cas12a (*N* = 34 for crRNA 24, *N* = 30 for crRNA 20, *N* = 33 for crRNA 18) and W355A Cas12a (*N* = 34 for crRNA 24, *N* = 26 for crRNA 20, *N* = 27 for crRNA 24 mix). In box plots, whiskers indicate 90% and 10% extreme values, the length of the box indicate interquartile range, the inner line represents the median and the black small vertical bar is the mean of the population.

Example R-loop formation profiles at −7 pN nm using 1 nM WT LbCas12a–RNA are shown in Fig. 2c,d and Supplementary Fig. 3. Using smaller diameter beads improved the signal-to-noise (Methods) and revealed greater R-loop heterogeneity than was detectable previously by van Aelst et al.^16^. Each trace was fitted with a hidden Markov model (HMM) to identify the minimum number of discrete states that could describe the data and to calculate the state positions in turns and their relative probabilities (Extended Data Fig. 2). For example, in Fig. 2c four discrete states (S_2_, S_3_, S_4_ and S_5_) could be identified. We interpret S_5_ events as PAM-distal DNA breathing, as observed by Cofsky et al.^30^ (see below). Another discrete state (S_1_) representing a shorter R-loop was also observed (Supplementary Fig. 3b). For crRNA 20, S_2_ appeared longer, so was denoted as $\mathrm{S}_2^\prime$. Events where the turns returned to zero consistent with reversible full R-loop dissociation (S_0_; Fig. 2c,d) were observed with similar probabilities for both crRNAs (Extended Data Fig. 3).

**Extended Data Fig. 4:**
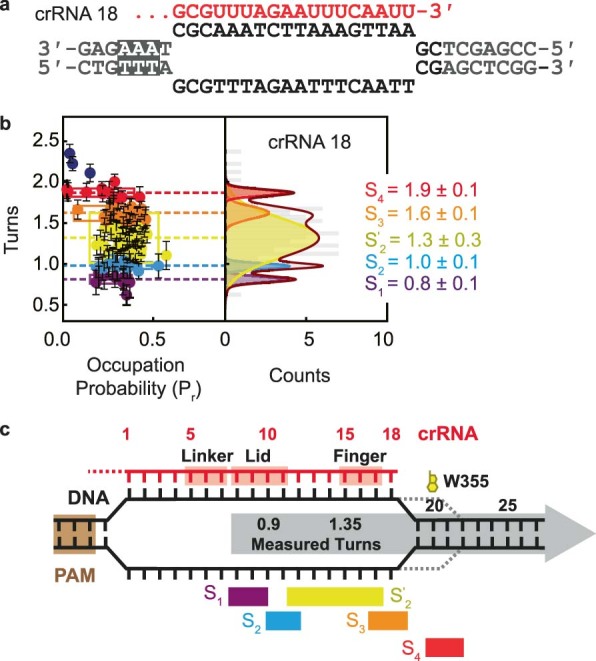
R-loop states for wild type Cas12a and crRNA 18 are consistent with a shorter R-loop. (**a**) Sequence of the crRNA 18. (**b**) Plot showing the five R-loop states identified by HMM analysis and their probability of occupation (P_r_) from multiple traces (*N* = 33), with state positions confirmed from Gaussian fitting. Box width corresponds to the full width at half maximum of each peak. Filled circles (left panel) represent mean values and error bars are s.d. of the measured turn size from HMM fitting of individual traces. Errors in turn values (right panel) are the s.d. from the Gaussian peak fitting. State S2’ (yellow) showed at intermediate position between S2 and S4. We interpret the S3 state as being the 18 bp R-loop while the S4 state is downstream DNA breathing. (**c**) Cartoon representation of the how each state compares to conformational checkpoints and possible R-loop sizes/DNA breathing. Boxes represent the approximate widths of the distributions. See main text and Fig. 2h for more details.

The occupation probability (*P*_r_) and average turn size of the states were calculated from multiple events (Fig. 2e,f and Extended Data Fig. 2). For each R-loop event, the maximum R-loop size was calculated (Fig. 2h). The mean and median R-loop sizes for crRNA 24 and crRNA 20 were similar. Using a crRNA with an 18-nucleotide spacer (crRNA 18), we observed a shorter average R-loop, consistent with expectations^34^ (Fig. 2h and Extended Data Figs. 2–4). We previously measured that the 20-bp R-loop of Cas12a corresponds to a change of 1.8 turns^16^. This matches S_4_. We therefore mapped the other states onto possible R-loop sizes (Fig. 2g). The S_1_, ${\mathrm{S}}_2/{\mathrm{S}}_2^\prime$ and S_3_ states could correspond to the Lid and Finger checkpoints that couple R-loop propagation to nuclease activation^14^ (Extended Data Fig. 1). HMM modelling suggests that R-loop dissociation can occur directly from these states. The S_5_ states can be estimated to correspond to an additional unwinding of ~2 bp from the S_4_ state. Although we cannot rule out that the change in turns partly corresponds to a change in writhe, this additional unwinding is consistent with previous breathing observations^30^. Breathing could also be inferred using crRNA 18 (S_4_ events in Extended Data Fig. 4). The similarity in R-loop distributions and overall occupancy of the S_5_ state for crRNA 24 and crRNA 20 suggest that a complementary 3′ overhang does not anneal and stabilise the downstream breathing events, favouring the model in Fig. 1c.

### Downstream DNA breathing stabilised by crRNA binding

**Fig. 3:**
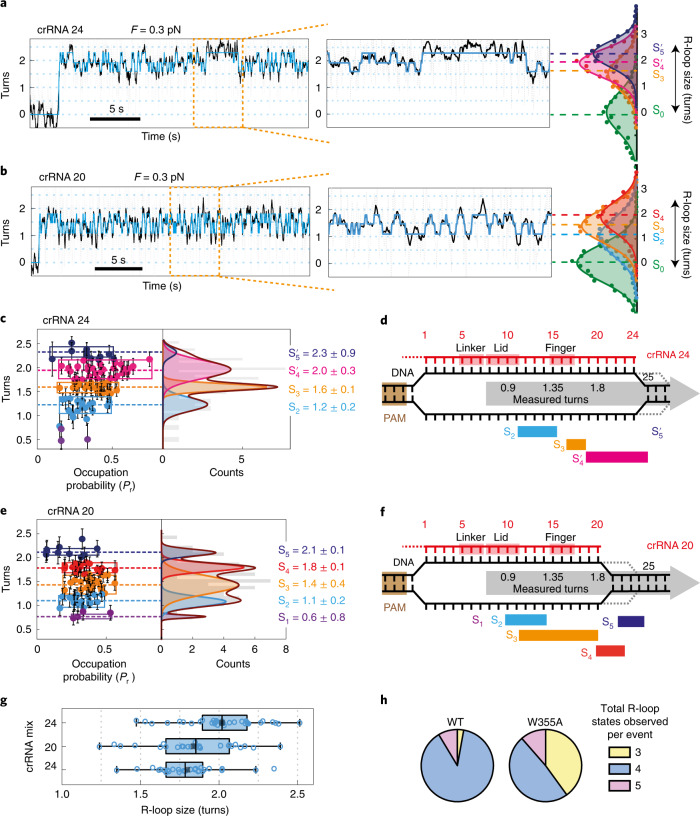
Mutation of the W355 allows annealing between the 3′ end of the crRNA and the targeted strand during downstream breathing. **a**, Example R-loop formation trace at −7 pN nm with W355A Cas12a and crRNA 24 showing hopping between three states identified by HMM fitting (blue); S_3_, $\mathrm{S}_4^\prime$ and $\mathrm{S}_5^\prime$. **b**, Example R-loop formation trace with W355A Cas12a and crRNA 20 showing hopping between three states identified by HMM fitting (blue); S_2_, S_3_ and S_4_. **c**,**e**, Plots for crRNA 24 (**c**) and crRNA 20 (**e**) showing the R-loop states identified by HMM analysis and their probability of occupation (*P*_r_) from multiple R-loop formation traces (*N* = 34 for crRNA 24, *N* = 26 for crRNA 20), with state positions confirmed from Gaussian fitting. Box width corresponds to the full width at half maximum of each peak. Filled circles (left) are mean values and error bars are standard deviation in turn positions measured from HMM fitting of individual traces (Methods). Errors in turn values (right) are standard deviation from the Gaussian peak fitting. **d**,**f**, Summary of the R-loop states classified from crRNA 24 (**d**) and crRNA 20 (**f**) alongside the crRNA contact positions of the linker, lid and finger^14^ (Extended Data Fig. 1). Dotted lines represent transient downstream breathing events that produce the extended states (S_5_ or $\mathrm{S}_5^\prime$). **g**, Box plots comparing main R-loop sizes measured from HMM analysis for crRNA 24 (*N* = 34), crRNA 20 (*N* = 26) and crRNA 24 mix (*N* = 27) (Extended Data Figs. 6 and 7). Whiskers indicate 90% and 10% extreme values, the length of the box indicates interquartile range, the inner line is the median and the black small vertical bar is the mean of the population. **h**, Pie charts of percentage of event traces showing 3, 4 or 5 states identified by HMM fitting for WT and W355A Cas12a events using crRNA 24 (Extended Data Fig. 2a and 6a).

**Extended Data Fig. 5:**
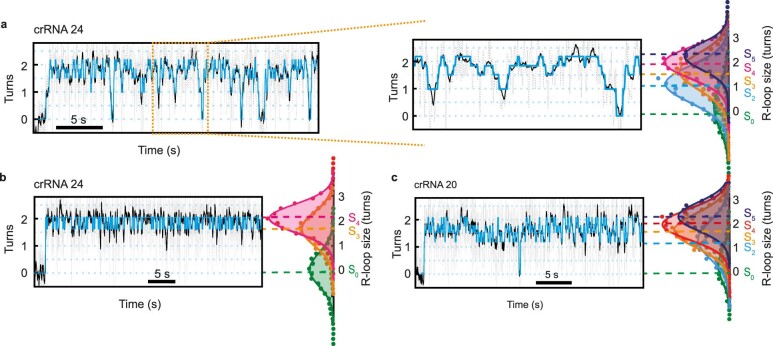
Example traces and event identification for W355A Cas12a using either crRNA 24 or crRNA 20. See main text and Fig. 3 for further details. See Extended Data Fig. 6 for turns states from individual events. (**a-b**) Example events for crRNA 24. In panel b, hopping was observed only between 2 states: S_3_ and S_4_. The slower R-loop formation at the start allowed clear identification of S_0_ without the necessity for an R-loop dissociation event. (**c**) Example events for crRNA 20.

**Extended Data Fig. 6:**
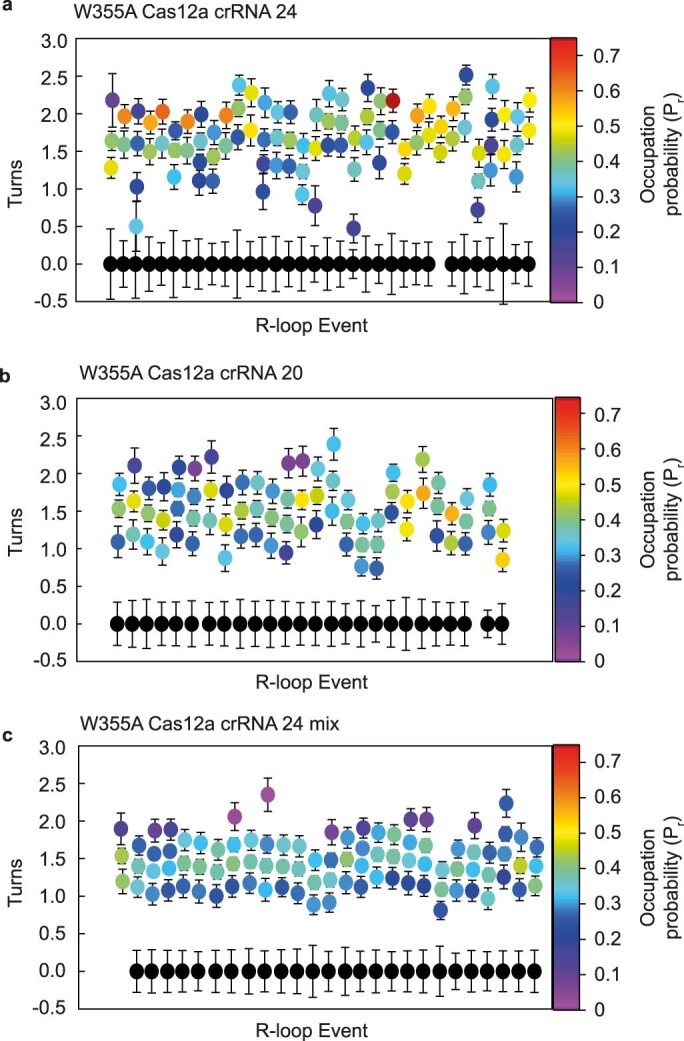
Size and dynamics of states for individual R-loop events at -7 pN nm using W355A Cas12a. For each R-loop formation event using 1 nM W355A Cas12a and crRNA 24 (**a**, *N* = 34 using 2 DNA molecules), crRNA 20 (**b**, *N* = 26 using 1 DNA molecule), or crRNA 24 mix (**c**, *N* = 27 using 1 DNA molecule), individual turn states were identified by HMM (Materials and Methods). The probability of occupancy (P_r_) of the fitted states was calculated and is shown for each state by the heat map. Filled circles represent mean and error bars are s.d. of the measured turn size from HMM fitting of individual traces. In a few events in all panels, the R-loop dissociated state (black) could not be identified as the R-loop formed immediately upon turning to negative torque.

To explore the role of the LbCas12a aromatic R-loop stacking residue, we mutated W355 to alanine and tested the R-loop dynamics using crRNA 24 and crRNA 20. Example events are shown in Fig. 3a,b and Extended Data Fig. 5. Using crRNA 24, W355A demonstrated greater occupancy of longer R-loop states (Fig. 3c) with fewer states identified by the HMM fitting per event (Fig. 3h and Extended Data Fig. 6); S_2_ was less frequently observed than with WT, while S_1_ was not measurably occupied. The most occupied state was 2.0 ± 0.3 turns (designated $\mathrm{S}_4^\prime$), which we suggest corresponds to a 20-bp R-loop plus 2 further unwound base pairs, equivalent to the transient S_5_ state of the WT enzyme (Fig. 3d). The additional $\mathrm{S}_5^\prime$ corresponds to 2.3 ± 0.9 turns, which we interpret as additional downstream unwinding by 2–3 bp not observed as a long-lived state with WT. R-loop dissociation events were observed, but less frequently than with WT (Extended Data Fig. 3).

**Extended Data Fig. 7:**
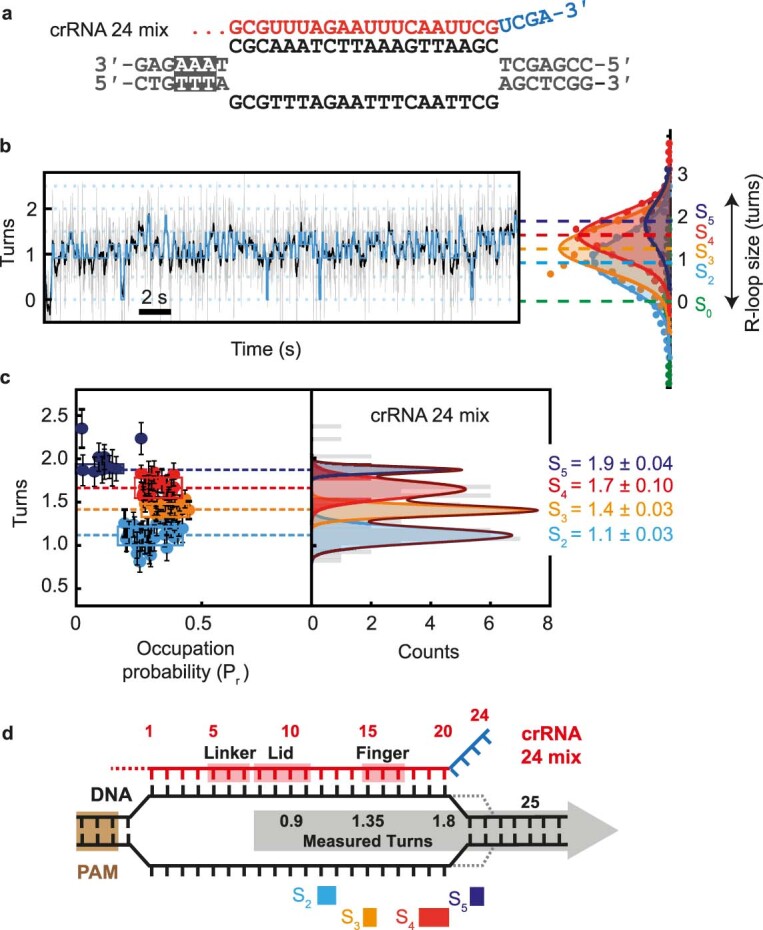
R-loop states for W355A Cas12a and crRNA 24 mix do not produce extended DNA breathing states. (**a**) Sequence of the crRNA 24 mix. The 3ʹ RNA extension (blue) is complementary with the NTS rather than the TS as in crRNA 24 (Fig. 2a). (**b**) Example event for crRNA 24 mix. (**c**) Plot showing the four R-loop states identified by HMM analysis and their probability of occupation (P_r_) from multiple traces (*N* = 27), with state positions confirmed from Gaussian fitting. Box width corresponds to the full width at half maximum of each peak. Filled circles (left panel) represent mean values and error bars are s.d. of the measured turn size from HMM fitting of individual traces. Errors in turn values are the s.d. from the Gaussian peak fitting. State S_1_ was not identified (see Extended Data Fig. 6 for turns states from individual events). (**d**) Cartoon representation of how each state compares to conformational checkpoints and possible R-loop sizes/DNA breathing. Boxes represent the approximate widths of the distributions. See main text and Fig. 3e for more details.

In contrast to crRNA 24, the states identified with crRNA 20 were more like those observed using WT (Fig. 3b–f and Extended Data Figs. 5c and 6b). The mean and median R-loop sizes for crRNA 24 and crRNA 20 with W355A were notably different (Fig. 3g). The stabilisation of $\mathrm{S}_4^\prime$ and $\mathrm{S}_5^\prime$ with crRNA 24 could be due to annealing between the additional 3′ RNA bases and the transiently unwound TS bases. To test this, we measured the R-loop dynamics with crRNA 24 mix with a 3′ end that was not complementary to the TS (Extended Data Figs. 6c and 7). The states identified, and the R-loop size distribution (Fig. 3g), were more similar to crRNA 20, with the main R-loop being ~20 bp with only occasional downstream breathing (S_5_). This data is therefore also consistent with the model in Fig. 1c; in the absence of the aromatic residue, downstream DNA breathing is not necessarily more frequent, but the unwound TS is not protected from interaction with RNA residues 3′ to 20-bp heteroduplex. Annealing produces an extended R-loop ($\mathrm{S}_4^\prime$) with DNA breathing extending even further downstream ($\mathrm{S}_5^\prime$).

### Effects of W355 and the crRNA 3′ end on DNA cleavage

**Fig. 4:**
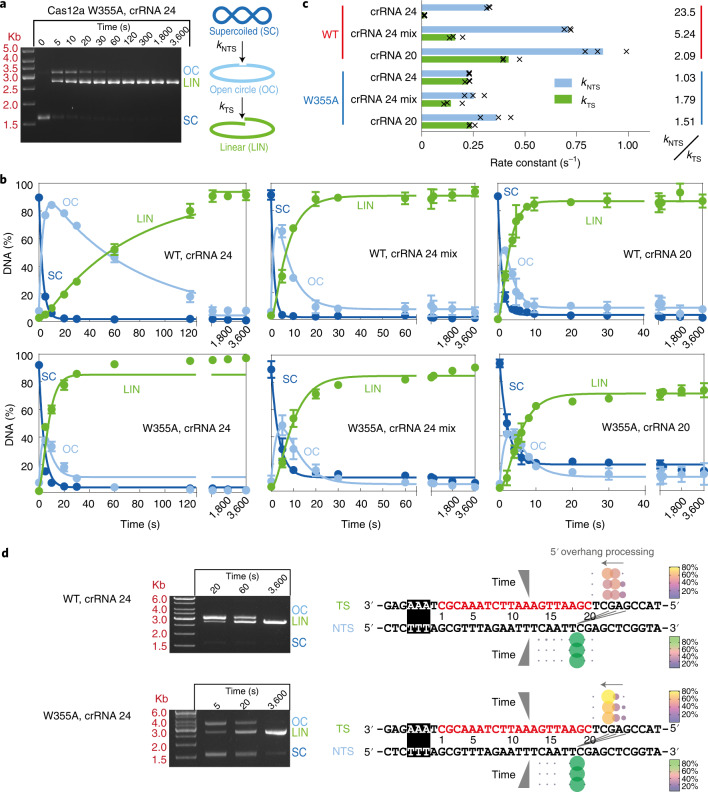
The targeted strand cleavage rate is affected by the nature of the crRNA 3′ end. **a**, Example agarose gel from three repeats of pSP1 cleavage time course using W355A Cas12a. Diagrams represent the supercoiled substrate (SC), nicked (open circle) intermediate (OC) and linear product (LIN) states, with the rate constants *k*_NTS_ and *k*_TS_ representing the ordered cleavage of the NTS followed by TS. **b**, Quantified data from individual cleavage time courses with WT or W355A Cas12a were simultaneously fitted using numerical integration to a simple cleavage model and the rate constants averaged^16^. Solid circles are means from three repeats with errors bars indicating standard deviation, while solid lines represent model simulations using the average parameters (**c**). **c**, Bars show mean rate constants for cleavage of the NTS and TS from three repeats (values shown as crosses). **d**, Agarose gel showing samples from the cleavage reactions used for nanopore sequence. pSP1 and either WT or W355A Cas12a were stopped at the times shown (chosen to be similar amounts of nuclease activity and based on the times from the repeats in **b**) and the cleaved ends of linear DNA from individual dsDNA cleavage events mapped using ENDO-Pore^41^. Circle diameter and colour both represent the relative percentage cleavage at that location on each strand (*N* = 3,064, WT 20 s; *N* = 5,812 WT 60 s; *N* = 5,597 WT 3,600 s; *N* = 3,063 W355A 5 s; *N* = 2,604 W355A 20 s; *N* = 3,984 W355A 5 s). Three diagonal lines represent the linkage between NTS and TS cleavage events for >92% of events. W355A produces the same cleavage loci as WT but the 5′–-3′ processing of the TS strand is faster (grey arrows). Note that ENDO-Pore returns cleavage loci of a single event that are closest to the 3′ end of each strand regardless of the order of cleavage (Supplementary Fig. 9).

If downstream breathing is necessary for docking the TS into the RuvC active site^14,30^, we reasoned that different occupancy of these states with different enzymes and crRNAs might influence nuclease activity. To measure DNA cleavage, we collected time points from an ensemble assay using a supercoiled plasmid DNA substrate where the nicked intermediate (open circle) and cleaved linear product can be separated and quantified by agarose gel electrophoresis^16^ (Fig. 4a; Methods). The cleavage profiles were fitted by numerical integration^16^ (Fig. 4b), to return rate constants for sequential NTS–TS cleavage (Fig. 4c). W355A appears less stable than WT (Supplementary Fig. 4), that may reduce the specific activity and explain the incomplete DNA cleavage in some instances (Fig. 4b).

As observed previously with WT Cas12a, crRNA 24 and a supercoiled plasmid^16^, the rate of TS cleavage was more than 23-fold slower than NTS cleavage (Fig. 4b,c). By contrast, using crRNA 20 or crRNA 24 mix, the TS rates are only twofold or fivefold slower, respectively. One hypothetical explanation for inhibition of TS cleavage with crRNA 24 is that W355 and REC2 have moved owing to structural transitions following NTS cleavage, allowing RNA–DNA annealing that inhibits TS docking similar to that proposed above with W355A and crRNA 24. The partial inhibition by crRNA 24 mix may indicate that a 3′ end also produces general steric inhibition and/or that there is annealing to the NTS that partly inhibits TS capture.

If R-loop extension by 3′ RNA–DNA annealing inhibits TS docking, we expected W355A to also be inhibited using crRNA 24 since this stabilises downstream breathing states before cleavage (Fig. 3). However, the TS and NTS rates were similar within error with the three crRNAs tested (Fig. 4a–c). The W355A mutation may be altering the REC lobe dynamics so that the steps towards TS capture are faster than annealing, bypassing the inhibitory states.

The dsDNA break locations were determined at a fixed time point using nanopore sequencing (Supplementary Figs. 5 and 8; Methods). The cleavage loci of the NTS and TS positions were similar in all cases, producing the expected 5′ overhang, but with differences in TS cleavage positions resulting in different overhang lengths. To explore this further, we mapped the cleavage generated by WT or W355A Cas12a with crRNA 24 at three time points (Fig. 4d). At each time point there were three principal cleavage loci on the TS, at positions 22, 23 and 24, producing 5′ overhangs of either 4, 5 or 6 nucleotides, respectively (Fig. 4d). There was a gradual shift from the 6-nucleotide to the 4-nucleotide overhang with time owing to slow 5′–3′ TS processing after initial cleavage^30^. Using W355A, the 5′–3′ processing of the TS was noticeably faster, with the majority product being the shortest 4-nucleotide overhang at the earliest time point (5 s).

### Downstream DNA clamping following non-target-strand cleavage

**Fig. 5:**
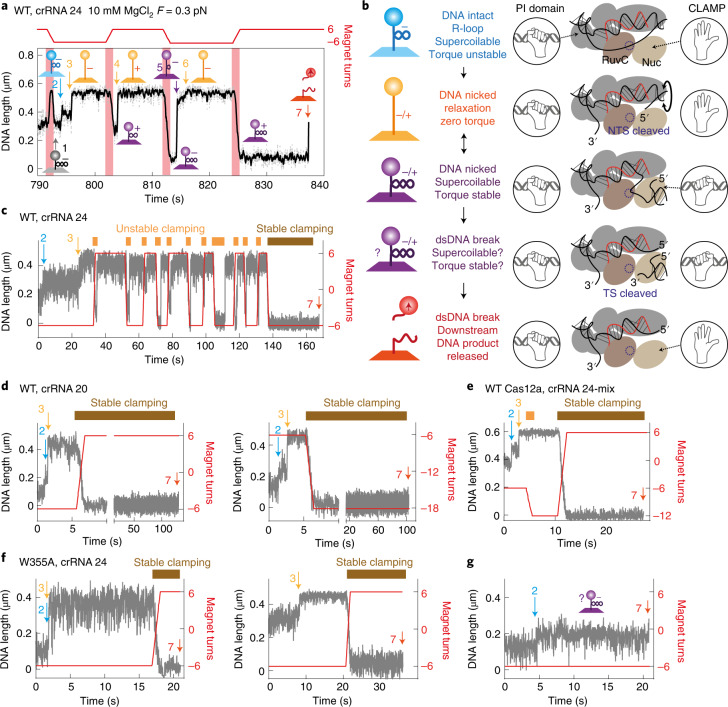
Cleavage of the non-targeted strand by Cas12a results in clamping of downstream DNA to produce a torque-stable state. **a**, Example time trace (grey, 60 Hz raw data; black, 10 Hz filtered) at 0.3 pN showing R-loop formation and DNA cleavage with WT Cas12a and Mg^2+^ ions. Magnet rotations from positive to negative values are shown in red. Numbered events are explained in the main text. The cartoons are also explained in **b**. **b**, Steps in the cleavage pathway. Cas12a is shown in cartoon form as in Fig. 1b,c. The effective topology of the DNA–bead tethers is shown in the cartoons (left). The hand cartoons represent clamped and unclamped states of the PI domain and, nominally, the Nuc. See main text for full explanation. **c**, Example WT trace with crRNA 24 showing formation of multiple torque unstable clamp states (yellow rectangles representing clamping time) before formation of an irreversible stable clamp state (brown rectangle). TS cleavage leading to bead loss (event 7) occurs at negative torque. See Extended Data Fig. 8a–c for further examples of cleavage at negative, positive and zero torque. **d**, Left and right, example WT traces with crRNA 20 showing immediate formation of the stable clamp state. See Extended Data Fig. 8d,e for further examples. **e**, Example WT traces with crRNA 24 mix showing formation of a single unstable clamp before the stable clamp state. See Extended Data Fig. 8f,g for further examples of cleavage at negative and positive torque. **f**, Example W355A traces with crRNA 24 showing immediate formation of the stable clamp state and cleavage at positive torque. **g**, Example W355A traces with crRNA 24 showing TS cleavage from the R-loop state without releasing supercoils following NTS cleavage, suggesting stable clamping before, or immediately upon, NTS cleavage.

To further explore DNA cleavage, we used a Mg^2+^-based buffer in the magnetic tweezers. We first examined WT Cas12a with crRNA 24 where we expected a delay in TS cleavage. DNA was positively supercoiled to inhibit R-loop formation before adding enzyme. The DNA was then rapidly unwound (<1 s) to produce negatively supercoiled DNA (event 1 in Fig. 5a), facilitating R-loop formation (event 2). Following NTS cleavage, free rotation at the nick would release negative supercoils, producing a further increase in apparent bead height to full length^36^ (event 3). Because of the limited time resolution of the assay, we could not identify whether this event was preceded by DNA breathing. Subsequent TS cleavage would produce a DNA double-strand break (DSB), breaking the bead–DNA tether and losing bead tracking. However, we noted unexpected properties of the nicked intermediate that are explained below and in a model in Fig. 5b.

Following event 3 in Fig. 5a, the tethered DNA should have been nicked and thus mechanical rotation of the magnets should not have had an effect. However, introducing twelve positive turns resulted in DNA length shortening owing to trapping of positive supercoils. After ~0.5 s, the DNA increased to full length in a single step (event 4), consistent with rapid free rotation at the nick^37^. When twelve negative turns were then introduced by magnet rotation, another reduction in bead height was immediately observed owing to trapping of negative supercoils. After ~1 s, the DNA length increased to close to full length in a rapid step (event 5). The remaining trapped negative supercoils were more gradually released over ~2 s (event 6). Finally, introduction of twelve positive turns produced positively supercoiled DNA. After a delay at ~11.5 s, loss of bead tracking (event 7) indicated a DSB in the DNA–bead tether.

These observations can be explained by Cas12a transiently clamping the downstream DNA following NTS cleavage to trap the R-loop and DSB in an isolated topological domain (Fig. 5b). Before NTS cleavage, the PAM-proximal DNA end is clamped by the PI domain but the PAM-distal downstream DNA is free to rotate. Upon NTS cleavage, free rotation of the downstream DNA reduces the torque to zero. Cleavage also results in a conformation change in REC2 that is coupled to inward motion towards Nuc^14^. We suggest that this results in the downstream DNA being clamped by Nuc so that it can no longer rotate. As the DNA nick is now held within a topological domain, magnet rotation can trap supercoils in the rest of the DNA. This double-clamped state is in dynamic equilibrium with the single-clamped state, so trapped supercoiling may again release by free rotation at the nick after a delay. In the double-clamped state, the TS is held close to the RuvC active site and cleavage can occur, but double-clamping may prevent immediate product dissociation. Once the Nuc clamp releases the DNA, the downstream product will be released. This is consistent with previous experiments showing that Cas12a retains interaction with the PAM-proximal product and releases first the PAM-distal DNA^13,15,17,23,25^. Importantly, because both DNA relaxation following NTS cleavage and bead loss following TS cleavage will only be observed if the Nuc clamp is open, observed lifetimes do not necessarily correspond solely to the chemical strand breakage rates but could also reflect the stochastics of the clamp opening and closing.

**Extended Data Fig. 8:**
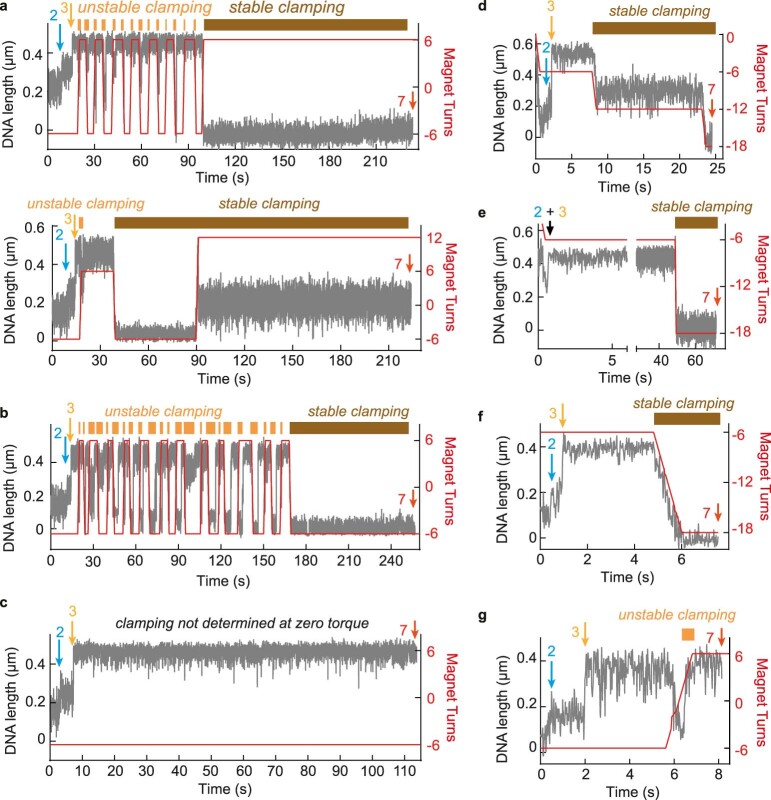
Examples of single-molecule DNA cleavage by WT Cas12a using crRNA 24, crRNA 20 and crRNA 24 mix. Grey lines show raw data at 60 Hz. Enzyme was introduced into the flow cell at positive torque to avoid R-loop formation. After turning the magnets to – 6 turns (-7 pN nm, red line), R-loop formation (event 2) and NTS cleavage (event 3) were observed. Magnet turns were then switched between positive and negative torque to capture unstable, reversible clamping (orange boxes) or stable clamping (brown boxes). TS cleavage and product release led to loss of bead tracking (event 7). See main text for full details. (**a**) Two cleavage events with crRNA 24 where event 7 occurred at positive torque showing either multiple unstable states (*upper graph*) or only a single unstable state (*lower graph*) before the stable clamp was formed. (**b**) Cleavage event with crRNA 24 where event 7 occurred at negative torque showing multiple unstable states before the stable clamp was formed. (**c**) Cleavage event with crRNA 24 where event 7 occurred at zero applied torque (where we cannot assign clamp states). (**d,e**) Cleavage events with crRNA 20 where event 7 occurred at negative torque. (**f**) Cleavage event with crRNA 24 mix where event 7 occurred at negative torque. (**g**) Cleavage event with crRNA 24 mix where event 7 occurred at positive torque.

In Fig. 5a, the DSB is produced from the clamped state at positive torque. Figure 5b shows another example using WT Cas12a and crRNA 24 where the DSB is produced from a longer-lived stable clamp state at negative torque and is preceded by multiple unstable clamp states. Further examples in Extended Data Fig. 8a,b show that the DSB is always produced from a hyperstable torque-resistant clamp state and is preceded by multiple unstable clamp states. In the ensemble cleavage assays (Fig. 4), there would not be any applied torque following NTS cleavage. Accordingly, dsDNA cleavage was also observed in the tweezers at zero torque (Extended Data Fig. 8c).

DNA cleavage in the tweezers was then tested using WT Cas12a and crRNA 20 (Fig. 5d and Extended Data Fig. 8d,e) or crRNA 24 mix (Fig. 5e and Extended Data Fig. 8f,g). DSBs were again produced from stable clamped states at either positive or negative torque. For crRNA 20 the stable clamped state was immediately formed without producing unstable intermediates. For crRNA 24 mix, formation of an unstable state was observed but the stable clamp state was more quickly formed than with crRNA 24. The time taken to establish the stable clamp state is therefore shorter for crRNA 20 or crRNA 24 mix and correlates with the faster rates of TS cleavage (Fig. 4b,c). The slower TS cleavage with crRNA 24 must therefore be due to unstable clamp states that delay formation of the stable state necessary for TS cleavage.

**Extended Data Fig. 9:**
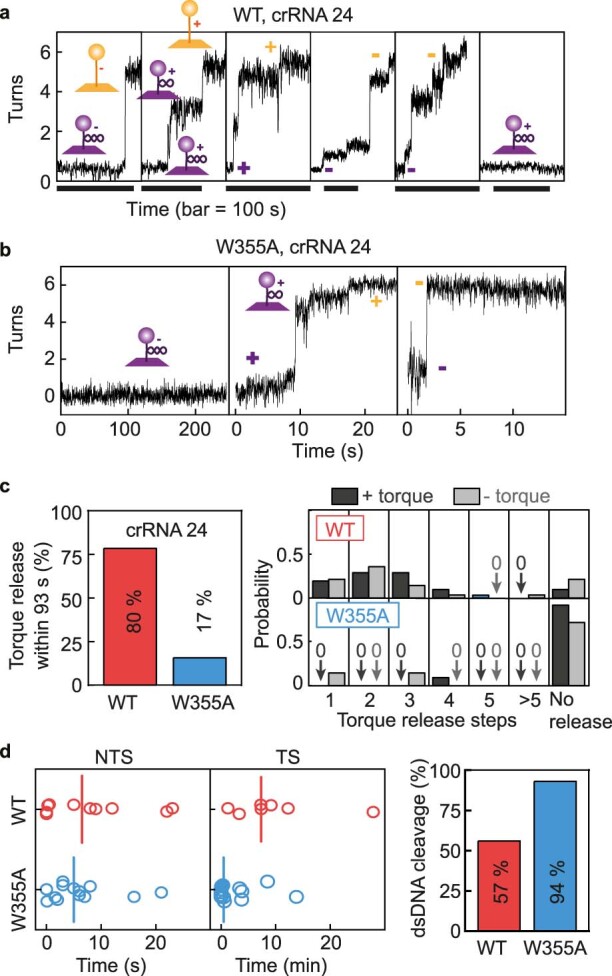
Probabilities of steps in supercoil release and DNA cleavage during clamped states. Clamped states for WT (**a**) and W355a (**b**) with crRNA 24 (10 Hz filtered). For WT cas12a, supercoiling release occurred in steps, the size and lifetime of which varied stochastically. For W355A, most events were stable (clamped states) over hundreds of seconds with occasional release events observed from unstable states. (**c**) (*left panel*) Percentage of clamped events at positive or negative torque that released (N = 50/63 for WT, N = 3/18 for W355A. *p* = 0.00001, using one tailed two proportion z-test the result is significant at *p* < 0.05). Note for W355A, most clamped events resulted in TS cleavage (*right panel*). Number of release steps from clamped to relaxed states at +7 pN nm (blue) or -7 pN nm (grey). Where the clamped state did not release, the average waiting time was 93 ± 20 s. For WT, the median release steps was 2 regardless of the torque sign. Steps release was similar for W355A but with insufficient events to reliably determine the median steps. (**d**) Scatter plots for WT (red) and W355A (blue) showing time between R-loop formation and DNA relaxation (NTS cleavage, *left panel*) and between DNA relaxation and loss of bead tracking (TS cleavage, *middle panel*). Lines are medians (*right panel*). Bar plot showing DSBs for WT (N = 8/14) and W355A (N = 16/17, *p* < 0.00001, using one tailed two proportion z-test the result is significant at *p* < 0.05). For DNA that only showed NTS cleavage, TS cleavage was not observed after an average time of 26 minutes. TS cleavage measured from bead release was faster for W355A, although there were fewer measurable WT events over a reasonable experimental timescale; 57% of molecules for WT over the experimental timescale compared to 94% for W355A.

With W355A and crRNA 24, rapid formation of the stable clamped state and DSB production was observed (Fig. 5f,g). While the majority of WT Cas12a cleavage events with crRNA 24 resulted in initial clamp states that released torque in 1–5 steps (Extended Data Fig. 9a), W355A only infrequently produced an unstable clamp (Extended Data Fig. 9b,c). Consistent with the ensemble data (Fig. 4a–c), faster formation of the stable clamp reduced the time until bead loss (Extended Data Fig. 9d). In the data in Fig. 5g, dsDNA cleavage occurs from the R-loop state without forming a relaxed intermediate (that is, event 3 was not observed). This suggests that the stable clamp was formed before torque could be released following NTS cleavage. The clamp could occasionally form simultaneously with, or before, the cleavage step. Rapid formation of the stable clamp by W355A may explain why the 3′ RNA does not inhibit the reaction as observed with the WT enzyme.

## Discussion

**Extended Data Fig. 10:**
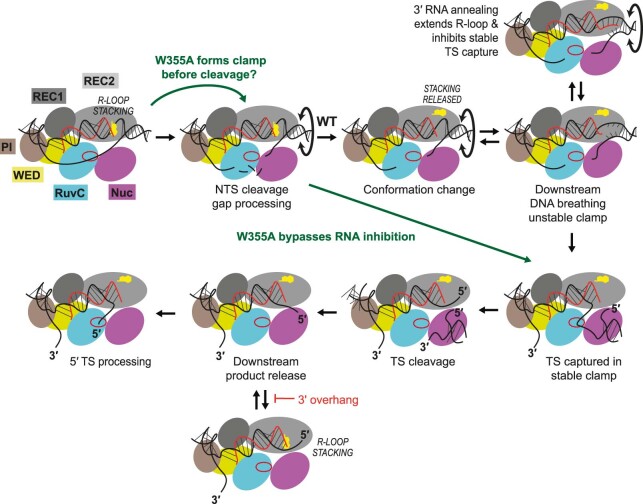
Model for sequential NTS and TS cleavage by Cas12a. Cas12a cartoon as in Supplementary Fig. 1. Full R-loop formation activates RuvC and the released NTS can enter the active site. NTS cleavage and processing of the ends can form a gap. For W355A Cas12a, the clamp (nominally the Nuc domain) may already have captured downstream DNA. NTS cleavage allows free rotation of the DNA end and torque release. For WT Cas12a, NTS cleavage triggers W355 movement so that R-loop stacking is released, allowing the clamp to capture and rearrange the downstream DNA to deliver the TS to the active site. However, if the crRNA has a 3ʹ overhang complementary to the downstream TS, movement of W355 allows the RNA to anneal. Clamp capture is inhibited, and free rotation of the DNA end and torque release can occur. This state is in dynamic equilibrium with an unstable clamped state that can stochastically enter a stable clamp state. For the W355A Cas12a, the absence of the stacking interaction allows immediate entry to the stable clamp state, bypassing the inhibition of any 3ʹ overhang. For both enzymes, TS cleavage can occur once the stable clamp state has formed. Following downstream product release and retention of the PAM proximal DNA end, further processing of the 5ʹ end of the cleaved TS can occur but this is inhibited by W355 stacking against the R-loop, while a 3ʹ RNA overhang may keep the 5ʹ TS end near the RuvC active site by inhibiting the stacking interaction and due to steric effects.

We explored R-loop dynamics and activation of NTS and TS cleavage by Cas12a using a combination of single-molecule assays, ensemble DNA cleavage, single cleavage event mapping by nanopore sequencing (ENDO-Pore) and single-molecule cleavage. Our results imply that a conserved aromatic residue (W355) in the REC2 subdomain that stacks against the end of the R-loop (Fig. 1a) prevents R-loop extension during downstream DNA breathing (Fig. 1c). Our results also indicate that a crRNA with a 20-nucleotide spacer facilitates faster target cleavage than a crRNA with a 24-nucleotide spacer as typically found in type V-A CRISPR loci. Mutation of the aromatic residue (W355A) resulted in faster target cleavage. To explain our data, we propose a modified DNA cleavage model (Extended Data Fig. 10). The model accounts for our observations of a clamped state formed following NTS cleavage that is necessary for TS cleavage, and which can be inhibited by annealing of the crRNA 3′ end. It remains to be determined why the 24-nucleotide spacer length and aromatic residue are conserved in nature yet come at the cost of slower target cleavage.

Using a magnetic tweezers assay, we observed dynamic interchange between defined R-loops states before DNA cleavage that could be mapped to previously identified conformational checkpoints (Figs. 2g and 3f and Extended Data Fig. 1). The pathways of R-loop formation were heterogenous between different events (Extended Data Figs. 2 and 5). A kinetic analysis of AsCas12a was also consistent with readily reversible R-loop propagation that limited the cleavage rate^15^. We additionally observed reversible R-loop dissociation and DNA breathing downstream of the R-loop (Figs. 2 and 3). The latter is suggested to be a key activity in providing the single-strand TS that can be delivered to the RuvC nuclease active site^30^.

Notably, using W355A, stable R-loop and breathing states longer than those seen with the WT enzyme were produced but only when the crRNA had a 3′ overhang complementary to the TS sequence; these states were not seen in the absence of the overhang or with a non-complementary overhang. In Cas12a structures, stacking of the aromatic residue against the end of the 20-bp R-loop directs the 3′ end of the crRNA away from the DNA^20,22^ (although extended RNA overhangs are not resolved in the structures, presumably owing to flexibility). Thus, stacking by W355 may prevent the 3′ end of the crRNA from accessing the upstream single-stranded TS that is transiently produced during breathing. Conversely, the absence of stacking in W355A might allow the crRNA 3′ end and TS to anneal, stabilising longer R-loop states that can then support further DNA breathing owing to the inherent properties of the 3′ end of an R-loop^30^. W355A also produced fewer transitions between states that would not be expected if aromatic stacking against the terminal R-loop base pair played a significant role in R-loop stabilisation.

With WT Cas12a, the TS cleavage rate was inhibited by a complementary 3′ overhang but was not in the absence of the overhang or with a non-complementary overhang. When measuring single-molecule DNA cleavage, we observed post-NTS cleavage states that trapped DNA torque (Fig. 5). We interpret the clamped state as downstream protein–DNA interactions that are necessary for delivering the TS to the RuvC active site^14,24,30^. Using the complementary crRNA overhang produced reversible unstable clamp states that eventually produced a stable clamp state that supported TS cleavage. In contrast, in the absence of the overhang or with a non-complementary overhang, the stable state was formed almost immediately, and TS cleavage was faster. We hypothesise that following NTS cleavage, movement of the REC2 subdomain^24^, and thus release of W355 stacking, allows access to downstream unwound DNA. If present, a 3′ RNA overhang complementary to the TS could then reversibly anneal, preventing full clamping until the free TS state is successfully captured (Extended Data Fig. 10).

By contrast, with W355A the rates of TS cleavage were similar for all crRNA tested and the stable clamping state was not inhibited by the complementary 3′ overhang. The loss of stacking might be expected to have also demonstrated inhibition by downstream annealing. However, we also observed with W355A that clamping could occur quickly enough that DNA torque was not lost following NTS cleavage. This suggests that W355A can access the clamped state by a route that does not allow annealing or that an additional propensity for downstream DNA unwinding can overcome any annealing. A previous investigation of an equivalent R-loop stacking residue mutation of AsCas12a (W382A) using crRNAs with 4-nucleotide 3′ overhangs indicated a reduction in INDEL formation of ~40 or ~90%, depending on the guide target. It may be that steps other than the relative rates of cleavage are more critical to successful editing; for example, the mutation of the aromatic residue producing a less stable protein, as we observed in vitro (Supplementary Fig. 4).

Although the DNA cleavage loci were similar between proteins and crRNAs, the length of the 5′ TS overhang (influenced by cleavage position and/or subsequent end processing) was affected. The length of the overhang varied as crRNA mix 24 ≥ crRNA 24 > crRNA 20 (Supplementary Fig. 5). This suggests that the crRNA 3′ end may produce a steric block that keeps the unwound TS in closer proximity to the nuclease active site to allow processing. W355A produced shorter overhangs than WT (Fig. 4d and Supplementary Fig. 5). We propose that tryptophan stacking following cleavage by the WT enzyme^23^ may stabilise the TS in a position that prevents access to the active site (Extended Data Fig. 10). Differences in end processing may influence the nature of repaired ends produced when applied in gene editing.

Single-molecule, structural and molecular dynamics studies of Cas12a showed that REC2 and Nuc move towards each other upon NTS cleavage, contracting the groove between the TS and RuvC active site^13,14,24^. We interpret the clamped state observed after NTS cleavage (Fig. 5), as resulting from this motion. Equivalent auxiliary target nucleic-acid-binding (TNB) domains that help load the TS into the RuvC active site are found across the Type V family (for example, the Nuc domain for Cas12a and Cas12b or the target-strand loading domain for Cas12e^33,38^). Although they adopt distinct structures^39^, a general role for TNBs could be to act as a clamp necessary for TS delivery to the RuvC active site^13,33^. Engineering of TNB and/or REC2 subdomains may be a fertile ground for producing new type V enzymes with improved DNA cleavage properties, either by reducing off-target cleavage^7^ by rejecting TS cleavage or by favouring more open states that are necessary for *trans*-cleavage. A recent publication from Aldag et al.^40^ demonstrated that type II Cas9 that lacks an equivalent TNB also produces torque-stable states during DNA cleavage, suggesting that this may be a general feature of coupling stabilising R-loop structures during DNA cleavage cycles.

## Methods

### Protein production and ribonucleoprotein assembly

The W355A LbCas12a mutation was generated by overlap extension PCR (primers 5′-AGTAAAGACATTTTCGGTGAGGCGAACGTGATCCGTGACAAATGG-3′ and 5′-CCATTTGTCACGGATCACGTTCGCCTCACCGAAAATGTCTTTACT-3′) with pSUMOCas12a^16^. WT and W355A LbCas12a were expressed and purified as published previously^16^. crRNAs were synthesised and HPLC-purified by IDT (5′-UAAUUUCUACUAAGUGUAGAUGCGUUUAGAAUUUCAAUUCGAGCU-3′, crRNA 24; 5′-UAAUUUCUACUAAGUGUAGAUGCGUUUAGAAUUUCAAUUCGUCGA-3′, crRNA 24 mix; 5′-UAAUUUCUACUAAGUGUAGAUGCGUUUAGAAUUUCAAUUCG-3′, crRNA 20; 5′-UAAUUUCUACUAAGUGUAGAUGCGUUUAGAAUUUCAAUU-3′, crRNA 18). For ribonucleoprotein (RNP) complex assembly, 250 nM Cas12a and 250 nM crRNAs were mixed in buffer RB (10 mM Tris-Cl, pH 7.5, 100 mM NaCl, 10 mM MgCl_2_, 0.1 mM dithiothreitol, 5 μg ml^−1^ bovine serum albumin) supplemented with 1 U per 20 µl^−1^ SUPERase-In RNase Inhibitor (ThermoFisher) and incubated at 37 °C for 1 h.

### Single-molecule magnetic tweezers experiments

The magnetic tweezers experiments were performed using a commercial PicoTwist microscope equipped with a 60 Hz Jai CV-A10 GE camera and PicoJai (v2019) software, and the data was analysed using PlayItAgainSam (v2019)^35^. For flow cell preparation, glass coverslips (Menzel Gläser No.1, 24 ×60 mm × 160 µm) were cleaned in three repeated cycles of 1 h sonication in 1 M KOH and then acetone and were subsequently cleaned with milliQ water and dried using compressed air. The coverslips were kept enclosed in glass jars to keep out any moisture until needed. Flow channels were prepared as before. DNA molecules (a 2-kb section of pSP1) were tethered to 500-nm paramagnetic beads (Adamtech)^42^, and the glass coverslip of the flow cell via 100 µg ml^−1^ anti-digoxigenin (Roche) in phosphate buffered saline as previously described^16,34^. Topologically constrained DNA were identified from rotation curves at 0.3 pN and the rotational zero reference (Rot_0_) set. One nanomolar RNP was used for all measurements. The R-loop formation and dissociation experiments (Figs. 2 and 3) were performed in buffer SB (10 mM Tris-Cl, pH 7.5, 100 mM NaCl, 1 mM EDTA, 0.1 mM dithiothreitol, 5 μg ml^−1^ bovine serum albumin) at 25 °C while the single-molecule cleavage experiments (Fig. 5) were performed in Buffer RB at 25 °C. The 500-nm paramagnetic beads show a narrow distribution of apparent DNA extension states owing to the reduced Brownian noise (Supplementary Fig. 6a,b). Cas12a-dependent R-loop formation events have a distribution greater than the Brownian noise from the bead and Cas12a does not induce additional noise in the bead (Supplementary Figs. 6c,d and 7a–d).

### Hidden Markov modelling of R- loop size and dynamics

Individual R-loop formation traces were sorted out from the raw data (60 Hz) using custom-built Matlab code and were filtered to 10 Hz. The DNA extensions were converted into turn values by linear fitting of the constant torque region of each hat curve (10 turns per second) at negative torque (Supplementary Fig. 2). Each trace was then fitted with an HMM^43^. From long time series of observations, HMM determines the kinetics between different states defined by a transition matrix and the Gaussian signal of each state, that assign each point in the observation sequence the most likely state of the system at a given time^44^. Both with WT (Fig. 2) and mutant Cas12a (Fig. 3) traces, the best fitting HMM was determined by extracting the turn values for each state in the HMM model using custom-built Fortran code. The histograms of turn values of each state were then separately fitted with a Gaussian model (in Origin Lab 2020b; https://www.originlab.com/) to extract the state positions. The HMM that described state positions separated by >0.1 turns was considered as the best fit as lower values resulted in the merging of Gaussian peaks and poor fitting of the traces. Next, for each trace the occupation probability (*P*_r_) of the states was determined by calculating the ratio of the lifetimes of each state (using HMM) and the total time of the measured R-loop event. The rupture event probability was measured by counting the number of events showing transitions from the R-loop to basal state and dividing by the total time of the trace. The MATLAB and Fortran codes used for analysis are available upon request.

### Ensemble DNA cleavage assays

Five nanomolar tritiated pSP1 was pre-heated in buffer RB at 25 °C for 5 min. Reactions were started by addition of 50 nM Cas12a RNP and incubated for the time specified. The reaction was quenched by adding 0.5 volumes of STEB (0.1 M Tris, pH 7.5, 0.2 M EDTA, 40% (wt/vol) sucrose, 0.4 mg ml^−^^1^ bromophenol blue) and incubating at 67 °C for 10 min. Samples were separated by agarose gel electrophoresis on a 1.5 % (wt/vol) agarose gel in 1X TAE (40 mM Tris-acetate, 1 mM EDTA, 10 μg ml^−1^ ethidium bromide) at 2 V cm^−1^ overnight (16 h) and visualised by ultraviolet irradiation. DNA bands containing supercoiled, linear or open circle DNA were excised and placed into scintillation vials. Sodium perchlorate (0.5 ml) was added to each gel slice, and tubes were incubated at 67 °C for 2 h to melt the agarose. The vials were cooled to room temperature and 10 ml Hionic-Fluor Scintillation Cocktail (Perkin Elmer) added to each vial and shaken thoroughly. Each vial was counted in a Tri-Carb Trio 3100TR Liquid Scintillation Counter for 10 min. Where indicated, the cleavage data was fitted to the model described in Mullally et al.^45^ using numerical integration in Berkeley Madonna v8.3.18 (https://www.berkeleymadonna.com) and further analysed using Graph Pad Prism v8 (https://www.graphpad.com).

### ENDO-Pore linked end mapping of Cas12a DNA cleavage

See Supplementary Fig. 8 for the ENDO-Pore workflow. Cleavage reactions using RNP (WT and W355A with crRNA 24) and pSP1 were quenched at different time points, as above, and the DNA purified using a DNA Clean & Concentrator-25 kit (Zymo Research). End repair and dA tailing were performed using NEBNext Ultra II End Repair/dA-Tailing Module (New England Biolabs) and the DNA ligated with a dT-tailed chloramphenicol cassette, recording the cleavage position. OmniMAX 2 T1R *E. coli* cells (ThermoFisher) were transformed with the ligation reactions and single colonies selected using 34 μg ml^−1^ chloramphenicol. Cleavage event libraries were generated by scrapping and pooling >20,000 colonies followed by plasmid purification. Rolling circle amplification was then performed using 10 ng of the cleavage library as template with EquiPhi29 DNA Polymerase and exonuclease resistant random hexamers (ThermoFisher). Reactions were incubated for 2 h at 45 °C, heat inactivated for 10 min at 65 °C, and the DNA purified using AMPure XP beads (Beckman Coulter). Rolling circle amplification products were debranched using 10 units per microgram DNA of T7 Endonuclease I (New England Biolabs) for 15 min at 37 °C. The debranching reaction was stopped by incubating with 0.8 units Proteinase K (New England Biolabs) for 5 min at 37 °C. Debranched products were purified using AMPure XP beads followed by size selection using the Short Read Elimination XS kit (Circulomics). Samples were prepared for nanopore sequencing using the Ligation Sequencing Kit (SQK-LSK109) combined with the Native Barcoding Expansion kit and sequenced using R9.4.1 MinION cells and MinKNOW v20.10.3 software (Oxford Nanopore Technologies). Raw reads were basecalled and demultiplexed using Guppy v4.5.4 (Oxford Nanopore Technologies). DNA sequence data were filtered using NanoFilt^46^. Circular concatemeric sequences were generated using C3POa v2.2.2^47^. Individual dsDNA breaks on pSP1 were identified using sequences with ≥5 concatemer repeats using bespoke software (Cleavage Site Investigator v1.0.0)^41^.

### Statistical analysis

The statistical significance of differences in torque release and dsDNA cleavage percentages (Extended Data Fig. 9c,d) was calculated using one tailed two proportion *z*-test. Test results are mentioned as *P* values in the legends. In box charts, whiskers indicate 90% and 10% extreme values, the inner line represents the median, the length of the box indicate interquartile range and the black small vertical bar the mean of the population. The *N* values for number of events (that is, switching between positive and negative turns) are stated in each figure where relevant. Each experiment was carried out on one to two different DNAs.

### Reporting summary

Further information on research design is available in the Nature Research Reporting Summary linked to this article.

## Online content

Any methods, additional references, Nature Research reporting summaries, source data, extended data, supplementary information, acknowledgements, peer review information; details of author contributions and competing interests; and statements of data and code availability are available at 10.1038/s41589-022-01082-8.

## Supplementary information

Supplementary Information Supplementary Figs. 1–9.

Reporting Summary

## Data Availability

All data supporting the findings of this study are available in the main text and extended data figures. The raw data that support the findings of this study are available at the University of Bristol data repository, data.bris, at 10.5523/bris.xjhk6a0gza0q27imvnw9r7mb2.

## Code availability

All custom-built codes used for single-molecule analysis are available from the corresponding author upon request. The code for the CSI software used for cleavage mapping of the nanopore data is available at 10.5281/zenodo.5057043.
