# Supplementary material for: CRISPR–Cas12a-mediated DNA clamping triggers target-strand cleavage
Source: Nat Chem Biol. 2022 Jul 14;18(9):1014–22. doi: 10.1038/s41589-022-01082-8 (PMC9395263; doi:10.1038/s41589-022-01082-8)
Supplement: Supplementary file 1 — Supplementary Figs. 1–9. [file 41589_2022_1082_MOESM1_ESM.pdf]

nature portfolio

https://doi.org/10.1038/s41589-022-01082-8

Supplementary information

CRISPR–Cas12a-mediated DNA clamping triggers target-strand cleavage

In the format provided by the authors and unedited

Supplementary Information to:

CRISPR Cas12a mediated DNA clamping triggers target strand cleavage

Mohsin M. Naqvi[1], Laura Lee[1], Oscar E. Torres Montaguth[1], Fiona M. Diffin[1] and Mark D. Szczelkun[1,*]

[1]DNA-Protein Interactions Unit, School of Biochemistry, Faculty of Life Sciences, University of Bristol, Bristol, BS8 1TD, UK

*Correspondence: mark.szczelkun@bristol.ac.uk;

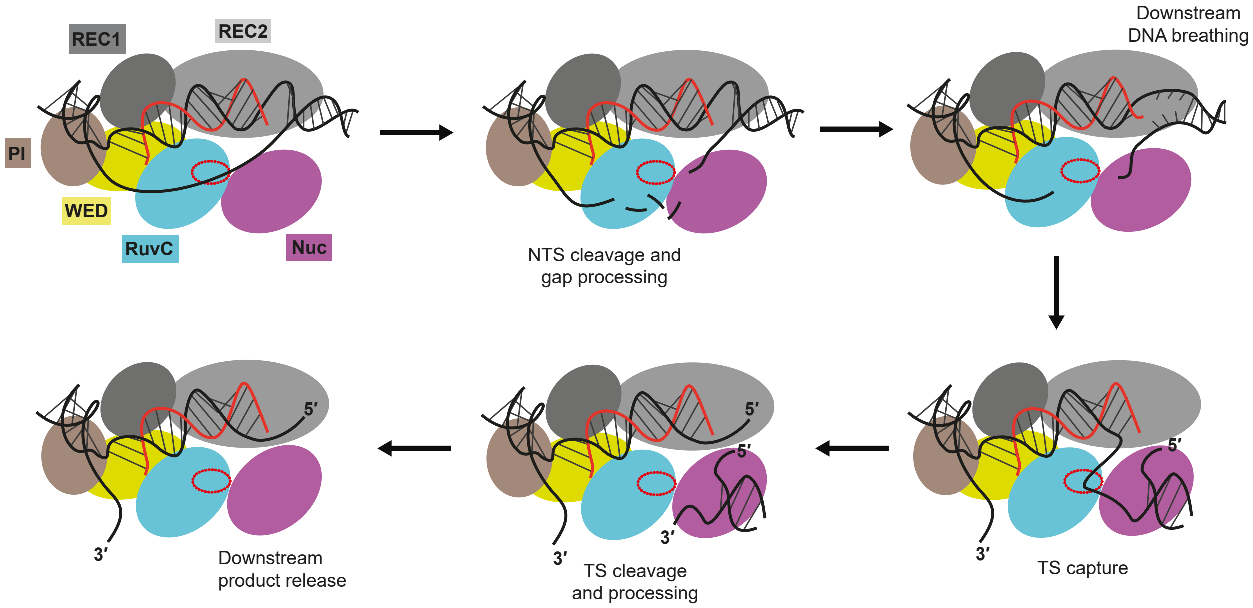

Supplementary Figure 1. Prevailing model for sequential cleavage of the NTS and the TS by Cas12a. Cartoon representation of Cas12a subdomains (see Fig. 1) and conformation changes necessary for sequential dsDNA cleavage. The RuvC active site is shown as a red oval. Prior to the first step, RNA:DNA hybrid formation has triggered the conformation checkpoints [14], resulting in lid opening that makes the catalytic pocket available for any ssDNA. The NTS is cleaved first because it is highly favoured to get in the pocket with the proper orientation. See main text for full details (also see Ref 13).

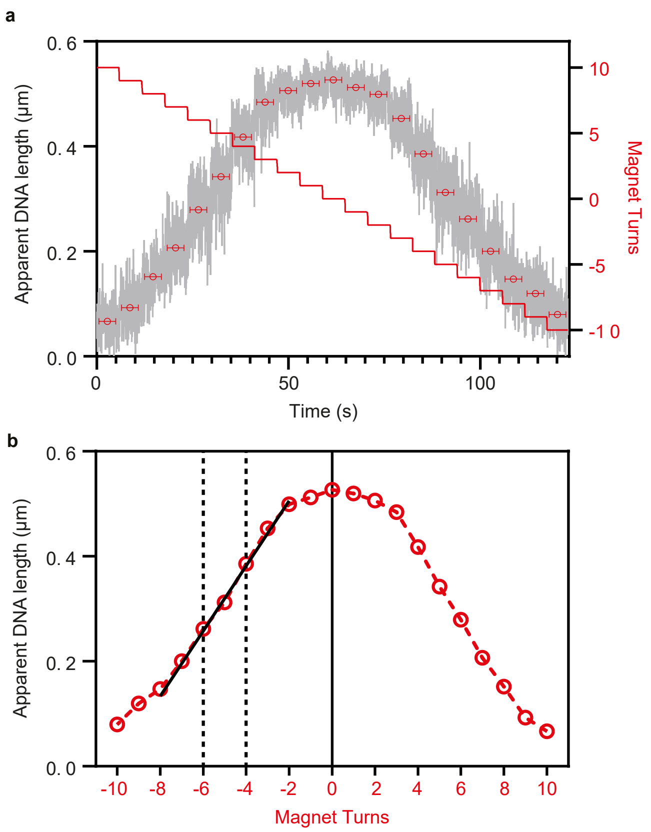

Supplementary Figure 2. DNA rotation-extension curve (Hat curve). (**a**) For each DNA-bead tether used, at the start of the experiment the apparent DNA length was recorded at 60 Hz using *F* = 0.3 pN (raw data, grey) for 5 s intervals at 1 turn increments from 10 to -10 magnet turns (red). The average apparent DNA length was calculated within a window (red horizontal bars) to allow for bead settling following each rotation [35]. (**b**) A straight-line fit (black) to the average lengths between -6 magnet turns (approximately the R-loop out position) to -4 magnet turns (approximately the R-loop in position) was used as a correction factor to convert DNA extension to a change in turns value. Note that the linear relationship extends below -6 and above -4, as shown.

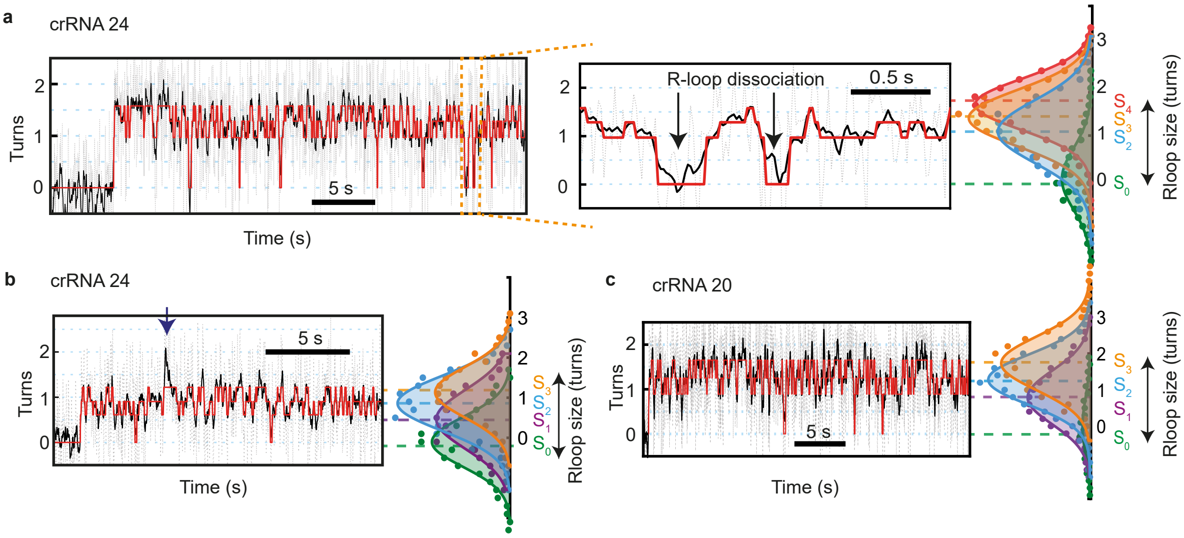

Supplementary Figure 3. Example traces and event identification for wild type Cas12a using crRNA 24 or crRNA 20. See main text and Fig. 2 for further details. See Extended Data Fig. 2 for turns states from individual events. (**a**) Example event for crRNA 24 showing full R-loop dissociation events which then reformed the S_2 state rapidly, suggesting that the Cas12a has not dissociated. (**b**) Example event for crRNA 24 where the full-length S_4 R-loop state does not appear to be accessed, although there is a transient increase that could be accessing S_4 and/or S_5 (blue arrow), but this could not be identified from the HMM analysis because of its infrequency during the measurement window. In general, there was great heterogeneity between events, both in the total turn sizes, number of states, and dynamics of the states (Extended Data Fig. 2). Here, the S_1 state was also identified. (**c**) Example event for crRNA 20. Here, the S_1 state was also identified.

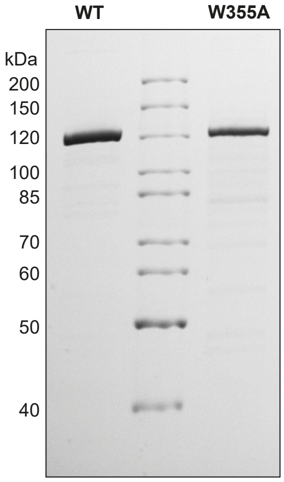

Supplementary Figure 4. LbCas12a preparations. 1 µg samples of WT and W355A LbCas12a following purification (Methods). SDS gel stained with colloidal blue (representative example from two separate preparations of each protein). All preparations followed the same protocol. Note that W355A showed a greater degree of breakdown products. The difference in stability possibly reflects changes in the packing of the subdomain that increases its flexibility and susceptibility to cellular proteases. However, we also noted a decrease in specific activity upon storage at -80 C that suggests the structure is more susceptible to unfolding/hydrolytic damage.

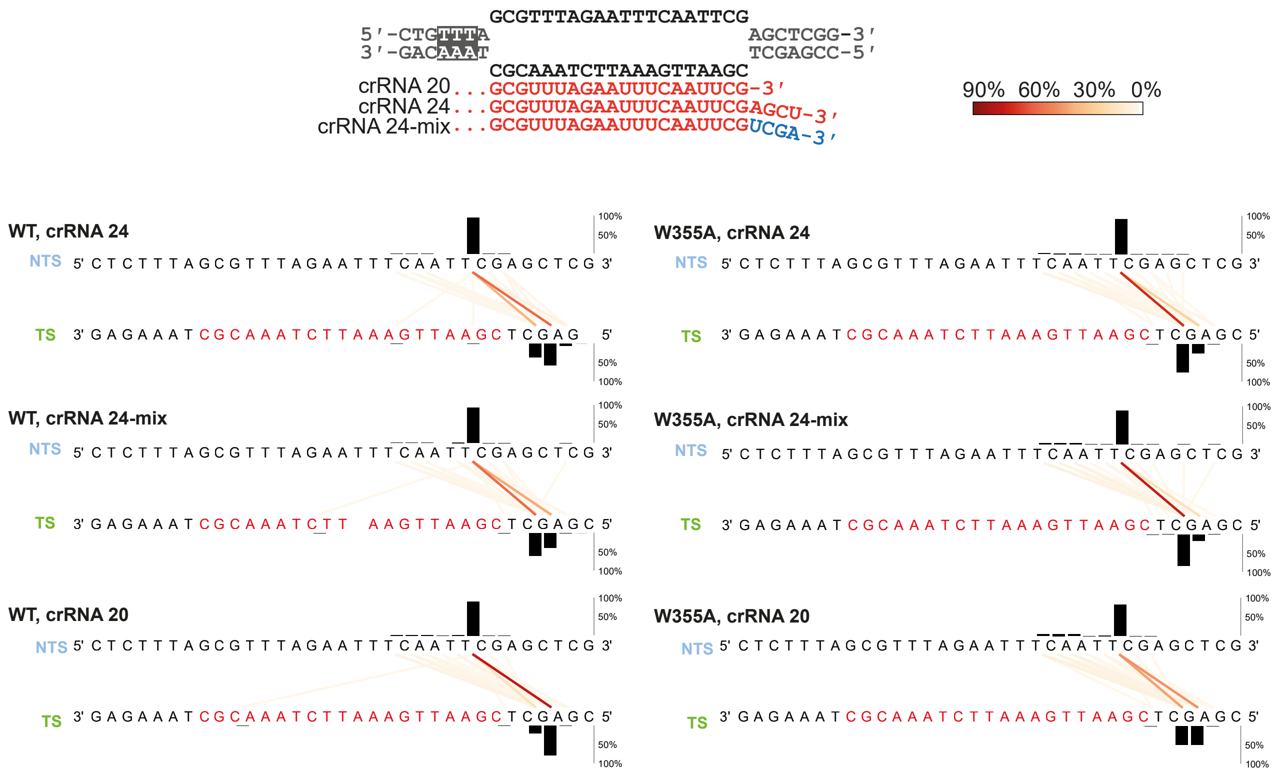

Supplementary Figure 5. Mapping of the sites of DNA cleavage by WT and W355A Cas12a at a fixed time point. Cleavage reactions using pSP1, WT or W355A Cas12a and crRNA 24, crRNA 24 mix or crRNA 20 were stopped at 5 min and the cleaved ends of the linear DNA from individual dsDNA cleavage events mapped using ENDO-Pore [36]. Data is presented as strand linkage plots; diagonal/vertical lines represent the linkage between NTS and TS cleavage loci for each event (coloured be percentage according to the heat map) while bar graphs above the NTS and below the TS indicate the percentage of cleavage at each loci regardless of the type of ends. (*N* = 1289, WT, crRNA 24; *N* = 2233, WT, crRNA 24 mix; *N* = 2094, WT, crRNA 20; *N* = 1654, W355A, crRNA 24; *N* = 1883, W355A, crRNA 24 mix; *N* = 1485, W355A, crRNA 20;). Note that ENDO-Pore returns cleavage loci of a single event that are closest to the 3′ end of each strand regardless of the order of cleavage (Supplementary Fig. 9).

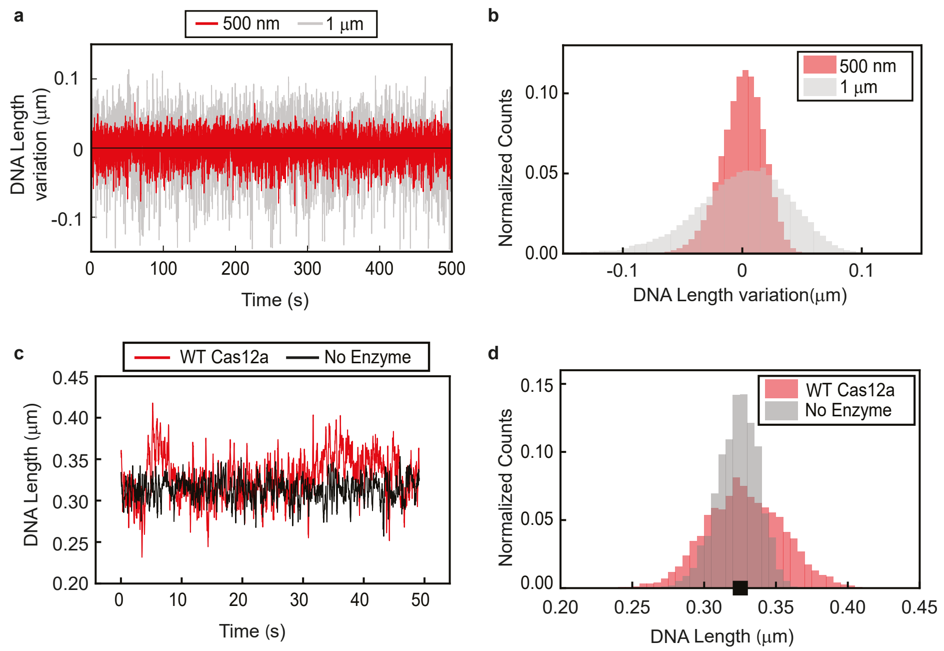

Supplementary Figure 6. Noise in the magnetic tweezers assay. (**a**) Time traces (filtered to 10 Hz) of apparent DNA length were collected for the 1 µm and 500 nm diameter beads. The variation in DNA length at – 4 turns (that corresponds to the R-loop formation conditions) was calculated from the average bead position. (**b**) Histograms of the DNA length variation values in panel a showing that the larger diameter bead is subject to greater Brownian noise [42]. (**c**) Comparison of times traces (filtered to 10 Hz) of DNA at -4 turns in the absence of enzyme and in the presence of enzyme after R-loop formation. (**d**) Histograms of the traces in panel c) showing that the fluctuations observed with Cas12a are greater than the background noise.

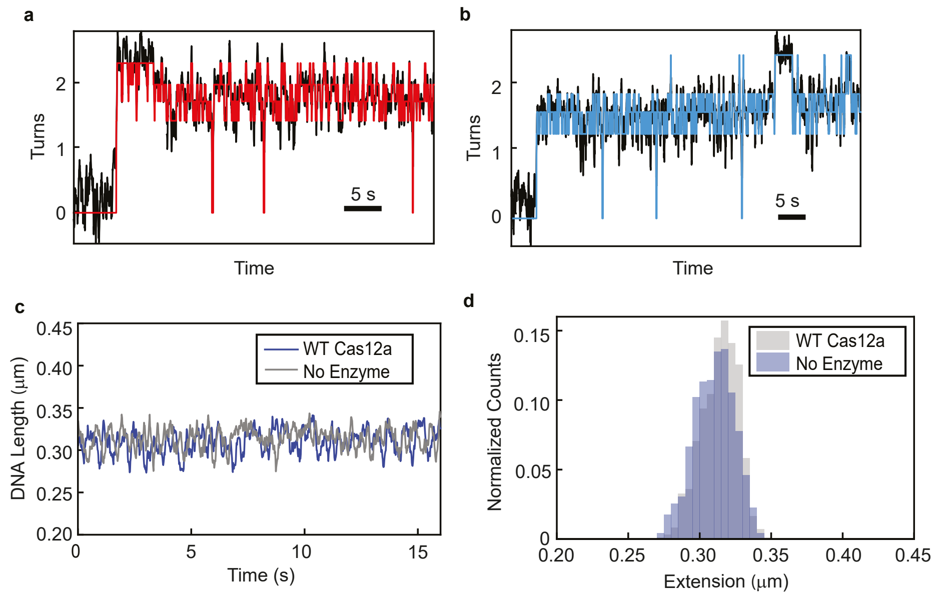

Supplementary Figure 7. The effect of enzyme concentration on R-loop formation and experimental noise. (**a, b**) Time traces (10 Hz) at -4 turns using crRNA 24 and either 0.8 nM wild type Cas12a (a) or 0.8 nM W355A Cas12a, showing the same dynamics as observed at 1 nM (Figures 2 and 3). (**c, d**) Time trace (c) and histogram (d) at +4 turns when the R-loop has dissociated. The noise is similar with or without enzyme, suggesting that Cas12a binding does not in general cause additional fluctuations. The single dissociation step results in the same noise as no enzyme that also suggests the dynamics at negative turns is due to single Cas12a binding.

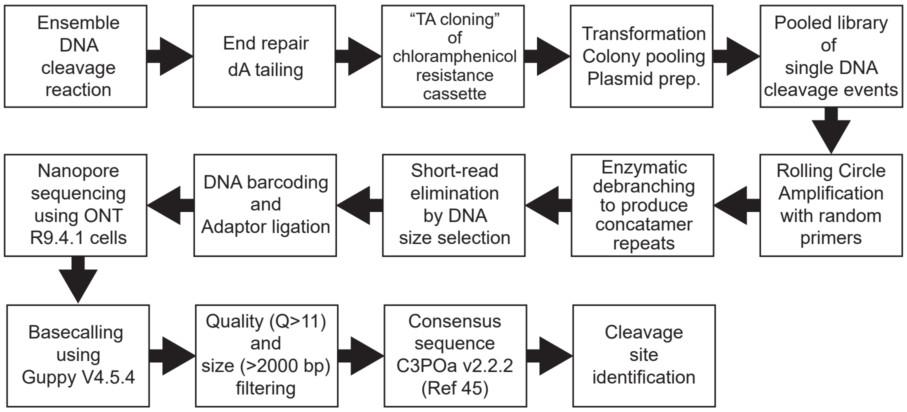

Supplementary Figure 8. ENDO-pore workflow. See Materials and Methods for details. Using DNA with ≥5 concatemer repeats produces consensus sequences with >99.5% median accuracy [36].

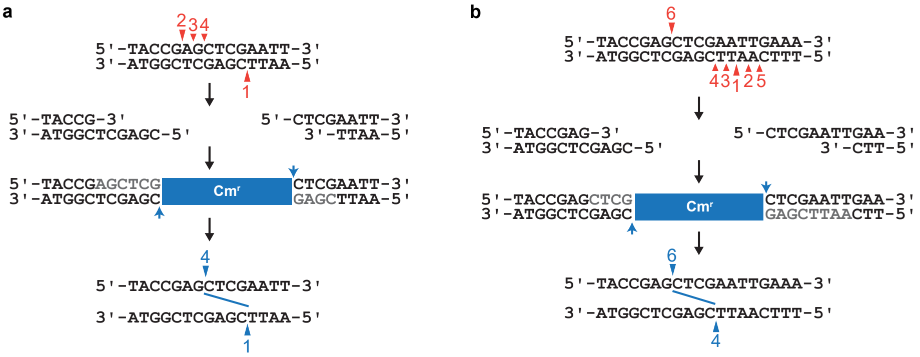

Supplementary Figure 9. The effect of end repair on the reported sites of DNA cleavage. When an endonuclease cuts at just one location on the top and on the bottom strand, ENDO-Pore reports the exact cleavage loci [36]. Following DNA cleavage and end repair, the ligated chloramphenicol resistance cassette (blue) is used to map the location of the cleavage sites (blue arrows): The 5′ end of the cassette provides the bottom strand cleavage position; the 3′ end of the cassette provides the top strand cleavage position. However, as observed for Cas12a (e.g., Ref 30), an endonuclease can make secondary cuts that further process the DNA ends. For example: (**a**) The bottom strand is cut at one location (event 1) but there is a subsequent 5′-3′ processing of the top strand (events 2→3→4); (**b**) Following the initial cleavage of the bottom strand (event 1), further random nicking 5′ and 3′ to the break produces a gap (events 2→3→4→5). The top strand is then cut at one location (event 6). In both cases, the cleavage sites reported are those closest to the 3′ ends of the respective strands and are independent of the order of the cleavage events (i.e., panel b).
