# Supplementary material for: CRISPR–Cas12a-mediated DNA clamping triggers target-strand cleavage
Source: Nat Chem Biol. 2022 Jul 14;18(9):1014–22. doi: 10.1038/s41589-022-01082-8 (PMC9395263; doi:10.1038/s41589-022-01082-8)
Supplement: Supplementary file 2 — Reporting Summary [file 41589_2022_1082_MOESM2_ESM.pdf]

nature portfolio

Corresponding author(s): Mark Szczelkun

Last updated by author(s): Jun 1, 2022

Reporting Summary

Nature Portfolio wishes to improve the reproducibility of the work that we publish. This form provides structure for consistency and transparency in reporting. For further information on Nature Portfolio policies, see our Editorial Policies and the Editorial Policy Checklist.

Statistics

For all statistical analyses, confirm that the following items are present in the figure legend, table legend, main text, or Methods section.

n/a	Confirmed	
☐	☒	The exact sample size (*n*) for each experimental group/condition, given as a discrete number and unit of measurement
☐	☒	A statement on whether measurements were taken from distinct samples or whether the same sample was measured repeatedly
☐	☒	The statistical test(s) used AND whether they are one- or two-sided<br>*Only common tests should be described solely by name; describe more complex techniques in the Methods section.*
☒	☐	A description of all covariates tested
☐	☒	A description of any assumptions or corrections, such as tests of normality and adjustment for multiple comparisons
☐	☒	A full description of the statistical parameters including central tendency (e.g. means) or other basic estimates (e.g. regression coefficient) AND variation (e.g. standard deviation) or associated estimates of uncertainty (e.g. confidence intervals)
☐	☒	For null hypothesis testing, the test statistic (e.g. *F*, *t*, *r*) with confidence intervals, effect sizes, degrees of freedom and *P* value noted<br>*Give P values as exact values whenever suitable.*
☒	☐	For Bayesian analysis, information on the choice of priors and Markov chain Monte Carlo settings
☒	☐	For hierarchical and complex designs, identification of the appropriate level for tests and full reporting of outcomes
☒	☐	Estimates of effect sizes (e.g. Cohen's *d*, Pearson's *r*), indicating how they were calculated

Our web collection on statistics for biologists contains articles on many of the points above.

Software and code

Policy information about availability of computer code

Data collection	Magnetic tweezers data was collected using commercial instrument using PicoJai software (v2019) supplied by Pico Twist. (Lionnet et al 2012, Cold<br>Spring Harb Protoc, 2012, 133-138.)<br>Nanopore sequences were collected using an Oxford Nanopore MiniON and the MinKNOW v20.10.3 software.
Data analysis	Magnetic tweezers data was analysed in PlayItAgainSam (v2019) supplied by Pico Twist. Data was analysed using Origin Lab 2020b (www.originlab.com/) and Graph Pad Prism v8 (www.graphpad.com/scientific-software/prism/)<br>The Fortran codes used for HMM analysis were published in Naqvi et al 2015 Biophys J, 109, 113-123.<br>DNA cleavage data was analysed using Graph Pad Prism v8 and by numerical integration in Berkeley Madonna 8.3.18 (www.berkeleymadonna.com) using the model described in https://doi.org/10.1093/nar/gkaa477.<br>Raw reads were basecalled and demultiplexed using Guppy V4.5.4 (Oxford Nanopore Technologies).<br>DNA sequence data were filtered using NanoFilt (github.com/wdecoster/nanofilt) (De Coster et al 2018 Bioinformatics, 34, 2666-2669).<br>The code for the CSI software (v1.0.0) used for cleavage mapping of the nanopore data is available at doi.org/10.5281/zenodo.5057043

For manuscripts utilizing custom algorithms or software that are central to the research but not yet described in published literature, software must be made available to editors and reviewers. We strongly encourage code deposition in a community repository (e.g. GitHub). See the Nature Portfolio guidelines for submitting code & software for further information.

Data

Policy information about availability of data

All manuscripts must include a data availability statement. This statement should provide the following information, where applicable:

- Accession codes, unique identifiers, or web links for publicly available datasets
- A description of any restrictions on data availability
- For clinical datasets or third party data, please ensure that the statement adheres to our policy

Example data for the single molecule magnetic tweezers and ensemble DNA cleavage assays are presented within the paper.
The full datasets that support the findings of this study are available at the University of Bristol data repository, data.bris, at https://doi.org/10.5523/bris.xjhk6a0gza0q27imvnw9r7mb2

Human research participants

Policy information about studies involving human research participants and Sex and Gender in Research.

Reporting on sex and gender	N/A
Population characteristics	N/A
Recruitment	N/A
Ethics oversight	N/A

Note that full information on the approval of the study protocol must also be provided in the manuscript.

Field-specific reporting

Please select the one below that is the best fit for your research. If you are not sure, read the appropriate sections before making your selection.

☒ Life sciences ☐ Behavioural & social sciences ☐ Ecological, evolutionary & environmental sciences

For a reference copy of the document with all sections, see nature.com/documents/nr-reporting-summary-flat.pdf

Life sciences study design

All studies must disclose on these points even when the disclosure is negative.

Sample size	No statistical methods were used to predetermine sample size. Sample sizes were based on previously published work (e.g. Szczelkun et al. 2014) and are standard for single molecule studies in the research field. Additionally, shown results were verified by statistical tests as indicated. For the single molecule magnetic tweezers assays, we analysed multiple R-loop formation state dynamics and then repeated this for N=26-34 events on 1-2 DNA molecules. For the DNA cleavage assays, these are ensemble assays that were repeated 3 times (replication, below). For the nanopore mapping experiments, we analysed 2604 - 5812 individual events depending on the dataset. Statistical significance of each finding was determined using one tailed two proportion z-test or two sample assuming unequal variance t-test. Test results were reported as p values in main text.
Data exclusions	In magnetic tweezers experiments, DNA molecules that showed no or incorrect supercoiling hat curves were excluded. Data was not excluded from the ensemble cleavage assays. For the nanopore mapping experiments, the data was filtered by quality factor and by number of repeats to ensure sufficient accuracy of the sequencing data.
Replication	All attempts at replication were successful.For the single molecule the R-loop dynamics measurements shown in the paper, the data represents hundreds of dynamic states measured across multiple R-loop events, on 1-2 DNA molecules. For single-molecule DNA cleavage experiments, each R-loop event led to DNA cleavage, so each cleavage event is a separate replicate. Test experiments on multiple DNA molecules were used to establish experimental conditions. For the ensemble DNA cleavage assays, the experiments were repeated independently 3 times.
Randomization	Samples were not randomized in the experiments. Randomization was not applicable as samples were allocated according to different conditions such as buffer conditions.
Blinding	Experiments were not blinded as the data acquisition and analysis were done in different conditions.

Reporting for specific materials, systems and methods

We require information from authors about some types of materials, experimental systems and methods used in many studies. Here, indicate whether each material, system or method listed is relevant to your study. If you are not sure if a list item applies to your research, read the appropriate section before selecting a response.

Materials & experimental systems

n/a	Involved in the study
☐	☒ Antibodies
☒	☐ Eukaryotic cell lines
☒	☐ Palaeontology and archaeology
☒	☐ Animals and other organisms
☒	☐ Clinical data
☒	☐ Dual use research of concern

Methods

n/a	Involved in the study
☒	☐ ChIP-seq
☒	☐ Flow cytometry
☒	☐ MRI-based neuroimaging

Antibodies

Antibodies used	Anti-digoxigenin antibodies; Cat. NO. : 11333089001; polyclonal antibody from sheep ; from Roche Diagnostics Germany.
Validation	The antibody was used without validation for the tethering of digoxigenin-labelled DNA to the glass coverslip and is a standard single molecule method (Lionnet, T. et al. Magnetic trap construction. Cold Spring Harb Protoc 2012, 133-8 (2012)). Manufacturer validation statement (https://www.sigmaaldrich.com/GB/en/product/roche/11333089001): The polyclonal antibody from sheep is specific to digoxigenin and digoxin and shows no cross-reactivity with other steroids, such as human estrogens and androgens.
